# High-throughput surface marker screen on primary human breast tissues reveals further cellular heterogeneity

**DOI:** 10.1186/s13058-021-01444-5

**Published:** 2021-06-13

**Authors:** Siru Virtanen, Reiner Schulte, John Stingl, Carlos Caldas, Mona Shehata

**Affiliations:** 1 CRUK Cambridge Institute, University of Cambridge, Cambridge, CB2 0RE UK; 2 Cambridge Institute for Medical Research, Cambridge University, Cambridge, CB2 0XY UK; 3 Cambridge Breast Unit, Addenbrookes Hospital, Cambridge University Hospital NHS Foundation Trust and NIHR Cambridge Biomedical Research Centre, Cambridge, UK; 4 Medical Research Council Cancer Unit, University of Cambridge, Hills Road, Cambridge, CB2 0XZ UK

**Keywords:** Normal breast, Surface markers, Breast epithelial cells, Stromal, Luminal progenitor, Antibody screen

## Abstract

**Background:**

Normal human breast tissues are a heterogeneous mix of epithelial and stromal subtypes in different cell states. Delineating the spectrum of cellular heterogeneity will provide new insights into normal cellular properties within the breast tissue that might become dysregulated in the initial stages of cancer. Investigation of surface marker expression provides a valuable approach to resolve complex cell populations. However, the majority of cell surface maker expression of primary breast cells have not been investigated.

**Methods:**

To determine the differences in expression of a range of uninvestigated cell surface markers between the normal breast cell subpopulations, primary human breast cells were analysed using high-throughput flow cytometry for the expression of 242 cell surface proteins in conjunction with EpCAM/CD49f staining.

**Results:**

We identified 35 surface marker proteins expressed on normal breast epithelial and/or stromal subpopulations that were previously unreported. We also show multiple markers were equally expressed in all cell populations (e.g. CD9, CD59, CD164) while other surface markers were confirmed to be enriched in different cell lineages: CD24, CD227 and CD340 in the luminal compartment, CD10 and CD90 in the basal population, and CD34 and CD140b on stromal cells.

**Conclusions:**

Our dataset of CD marker expression in the normal breast provides better definition for breast cellular heterogeneity.

**Supplementary Information:**

The online version contains supplementary material available at 10.1186/s13058-021-01444-5.

## Background

The human breast is a complex steroid-responsive organ which undergoes morphological and structural changes depending on the reproductive stage. The breast epithelium is composed of two known cell types, an outer layer of myoepithelial/basal cells and an inner luminal layer composed of separate secretory and hormone receptor-positive populations. These populations are organised into a series of ductal networks, surrounded by stromal cells and adipocytes [1–3]. This breast network is structured via a main stem or primary duct ending in a cluster of sac-like lobules termed terminal ductal lobular units (TDLUs). The origins and development of breast cancer revealed that most breast cancers originate from a single TDLUs [4]. Historically mammographic and histology analyses were limited in defining the exact cell compartment responsible for neoplastic transformation. Reliance on immunostaining for specific keratin (K) markers classifying breast cell types has led to discrepancy. K5 and K14 are often referred to as basal keratins based on their expression in the mouse mammary gland, specifically within the basal layer of the ducts, yet they were also expressed within the luminal layer of TDLUs of human breast tissues, therefore making cell identity difficult to interpret using these markers [5, 6]. A better understanding of the cellular heterogeneity existing in the breast epithelium and different cell states provides useful clues to how these cell types transform into the distinct breast cancer subtypes.

Many studies have relied on in vitro and in vivo assays to understand the hierarchical organisation and the progenitor/stem capacities of breast epithelial cells. One of the earliest studies used a combination of cell surface markers including EpCAM (ESA), CD10, CD49f (Integrin α6) and MUC1 (CD227) to identify the basal and luminal populations via flow cytometry [7, 8]. Subsequently, different cell isolation protocols and cell surface marker combinations were utilised to identify dissimilar subpopulations adding to the complexity with minimal overlap between studies [9–11]. Currently, the combination of two key cell surface markers, EpCAM and CD49f, are widely used as differentiation markers to identify the basal, luminal progenitor (LP), mature luminal (ML) and stromal compartments of the normal breast [12–14]. Investigating breast cellular heterogeneity has taken a leap forward with the enhancement of single cell omic studies. Single cell transcriptome analysis of primary human breast tissue confirmed the three main epithelial cell types and has highlighted that there are additional cell states within each cell population [15, 16]. The different cell states are essential to predicting a cellular trajectory hierarchy. Validating these novel cell states is problematic due to technical difficulties in isolating viable live cells based on their transcriptomic profile. The cell surface proteome is central to many biological functions which reflect cell fate, yet expression patterns of many cell surface markers in the human breast cell subpopulations are poorly defined.

Here, we identify specific CD marker expression patterns within the breast epithelium and stromal cell populations to generate a searchable dataset. We developed an analysis platform using standard flow cytometry and multiplexing for the simultaneous examination of epithelial and stromal cell populations. This protocol allowed us to identify and quantify the abundance of hundreds of CD markers on single cell suspensions of reduction mammoplasty specimens. Our data presents opportunities for new antibody panels that focus on stricter definitions of the cellular states of the human breast. Characterisation of CD proteins expressed by each breast subpopulation is informative as it will not only improve cell state classifications but may also provide insights into biological function.

## Methods

### Dissociation of human mammary tissue

All primary human materials were derived from reduction mammoplasties at Addenbrookes Hospital, Cambridge, UK, under full informed consent and in accordance with the National Research Ethics Service, Cambridgeshire 2 Research Ethics Committee approval (08/H0308/178) as part of the Adult Breast Stem Cell Study. All tissue donors had no previous history of cancer and were premenopausal (37–43 years old). Reduction mammoplasty specimens were transferred from the operating room on ice in sterile DMEM/F12 1:1 media (Invitrogen) supplemented with 5% FBS (Gibco/Invitrogen). Tissues were dissociated into single cell suspension as described previously [17]. Briefly, tissue was manually minced and incubated in DMEM/F12 1:1 medium with 10 mM Hepes plus 2% BSA, 5 μg/ml insulin (Invitrogen), 50 μg/ml gentamycin, 300 U/ml collagenase (Sigma) and 100 U/ml hyaluronidase (Sigma) with gentle shaking at 37 °C, overnight or for 16 h. Tissue fragments were harvested by washing with DMEM/F12 and spinning at 450*g* for 5 min at 4 °C. Fragments were triturated in trypsin-EDTA (0.25%; Stem Cell Technologies) for 2–3 min following a red blood cell lysis using ammonium chloride solution (Stem Cell Technologies). Cells were then washed in HBSS without calcium or magnesium, supplemented with 2% FBS, and centrifuged. Cells were then triturated in dispase 5 U/ml and 50 μg/ml DNase I for 1 min, followed by a final wash in HBSS plus 2% FBS and centrifuged.

### Surface protein screening using lyoplate technology

**Fig. 1:**
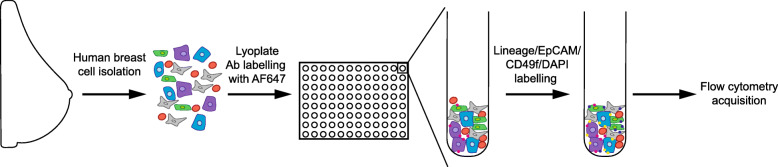
Schematic diagram depicting the experimental overview of the human breast surface protein marker antibody screen

Single cell suspensions from two human mammary reductions were pooled together and analysed using a commercial antibody screen, the BD Lyoplate™ Human Cell Surface Marker Screening Panel (BD Biosciences), containing AlexaFluor®647-conjugated antibodies with specificity for 242 cell surface markers and 9 isotype controls, arrayed across three 96-well plates. The cell surface marker antibody screen was performed twice using a total of 4 individual mammary reduction samples. 3–4 × 10^5^ breast cells were used for each antibody to ensure sufficient cells analysed to obtain a reliable positive signal. A detailed list of the antibodies can be found in Supplementary Table 1. Staining was performed as described by the manufacturer’s protocol with minor modifications. Briefly, the lyophilized antibodies were reconstituted with 110 μl of deionised water. One hundred microliters of breast cell suspension was aliquoted into three new 96-well plates at a density of 3–4 × 10^5^ cells/well. Twnety microliters of the reconstituted antibody was added to cells and incubated on ice for 20 min. The cells were then washed twice with HBSS plus 2% FBS and centrifuged at 300×*g* for 5 min to remove any unlabelled antibody. The cell pellet was incubated with the following primary antibodies: CD31-APC/Cy7, CD45-APC/Cy7, epithelial cell adhesion molecule (EpCAM)-PE, CD49f-PE/Cy7 (BioLegend). CD45 and CD31 were used to deplete contaminating haematopoietic and endothelial cells (collectively termed Lin+ cells). Cells were incubated with 4′,6-diamidino-2-phenylindole (DAPI, Invitrogen) before a final wash and data was acquired by flow cytometry using an LSR II flow cytometer (BD Biosciences) with a high-throughput sample attachment on the instrument, and 250,000–350,000 events per well were collected. The lyoplate workflow is shown in Fig. 1.

### Surface protein screening data analysis

Data analysis was accomplished using FlowJo v10 software (FlowJo LLC, Treestar, USA). The gating strategy (Figure S1) was designed to remove debris, dead and Lin+ cells. EpCAM and CD49f markers were used to discriminate between the basal, luminal progenitor, mature luminal and stromal cell types. To analyse each population for its AlexaFluor®647 positivity, a 1% positive events in the AlexaFluor®647 gate was the minimum criteria positive selection for each cell surface marker. Less than 1% event detections were deemed as negative cell surface markers and recorded as zero. Analytical data of percentage of AlexaFluor®647 positive events were exported to Excel and associated to sample ID, plate number row and column. To determine signal intensity, histograms were generated, and the control isotype median fluorescence intensity (MFI) was calculated using FlowJo. Bisector gating on the histogram was used to discriminate between positive and negative populations. Positivity was calculated as being 3 robust standard deviations of the control MFI. Selecting the positive population, the median, minimum and maximum fluorescence intensities were exported to Excel. Using the minimum and maximum values, variation in positive marker signal was categorised into 4 groups: 0 – < 1 log fluorescence intensity; 1 – > 1 and < 1.5 log fluorescence intensity; 1.5 – > 1.5 and < 2 log fluorescence intensity and 2 – > than 2 log fluorescence intensity.

### Aldehyde dehydrogenase (ALDH) flow cytometry assay

Human breast single cell suspensions were treated to detect the enzyme activity of aldehyde dehydrogenase (ALDH) using the Aldefluor Kit (StemCell Technologies) as per the manufacturer’s instructions. The cells were then preblocked with 10% normal rat serum (Sigma) and stained with the following antibodies: CD31-APC/Cy7 (Clone WM-59), CD45-APC/Cy7 (Clone HI30), EpCAM-PE, CD49f-PE/Cy7 (Clone GoH3) (all from BioLegend) in combination with one of the following antibodies CD140b-AF647 (Clone 28D4), CD142-AF647 (Clone HTF-1), CD26-AF647 (Clone M-A261), CD34-AF647 (Clone 581), CD340 (Her2)-AF647 (Clone Neu24.7), CD39-AF647 (Clone TU66), CD44-AF647 (Clone G44-26), CD49c-AF647 (Clone C3 II.1), CD66 (a,c,d,e)-AF647 (Clone B1.1/CD66), CD54-AF647 (Clone LB-2), CD55-AF647 (Clone IA10), CD13-AF647 (Clone WM15), CD73-AF647 (Clone AD2), CD15s-AF647 (Clone CSLEX1), CD151-AF647 (Clone 14A2.H1), CD166-AF647 (Clone 3A6), CD282-AF647 (Clone 11G7), CD63-AF647 (Clone H5C6), CD75-AF647 (Clone LN1), SSEA-4-AF647 (Clone MC813-70), TRA-1-81-AF647 (Clone TRA-1-81), CLA-Biotin-AF647 (Clone HECA-452), CD15-AF647 (Clone HI98) (all from BD Biosciences). Cells were then filtered through a 30-μm cell strainer and incubated with DAPI. Human cells were separated using an Influx cell sorter (Becton Dickinson). Single-stained control cells were used to perform compensation manually. Gates were set in reference to fluorescence-minus-one controls. The ALDH+ gate was set in reference to control populations incubated with the ALDH inhibitor DEAB in addition to Aldefluor. Flow cytometry data were analysed using FlowJo™ software.

### In vitro colony-forming assays

Flow-sorted human luminal progenitor cells were seeded into 60 mm plates with 2.5 × 10^5^ irradiated NIH-3 T3 feeder cells. The cultures were maintained in Human EpiCult-B (StemCell Technologies) supplemented with 5% FBS (StemCell Technologies) and 50 μg/ml gentamicin for 48 h and then the media changed to serum-free conditions and maintained for an additional 12 days. Colonies were fixed with acetone to methanol (1:1), stained with Giemsa (Fisher Scientific) and enumerated under a microscope.

### Statistical analysis

Data presented are the mean of multiple independent experiments and the standard error of the mean. One-way analysis of variance was used to test multiple groups followed by Tukey’s post-test to test significant differences between pairs of results. Comparisons between just two groups were analysed by t-test. Significance was set at *P < 0.05 and **P < 0.01.

## Results

To explore the heterogeneity of normal breast epithelial and stromal cells and to generate a dataset of surface protein expression, we subjected human reduction mammoplasty specimens to a panel of monoclonal antibodies specific for 242 human cell surface proteins using the BD Lyoplate system. Primary human breast tissue from two healthy donors per antibody screen was dissociated to single cells and pooled. Single cell suspensions were arrayed on the 96 well plates containing the AlexaFluor®647-conjugated lyoplate antibodies and controls. Subsequently, tagged cells were then subjected to the widely used flow cytometry staining protocol (Fig. 1). Flow cytometry (FC) analysis gating allowed the elimination of doublets, debris and endothelial/haematopoietic cells. The breast epithelial subpopulations and stromal compartments were then gated to identify negative and positive antibody markers (Figure S1a-b). The inclusion of the breast epithelial flow antibody strategy was imperative to eliminate the number of false positive surface markers irrelevant to the stromal/epithelial content of the normal human breast.

**Fig. 2:**
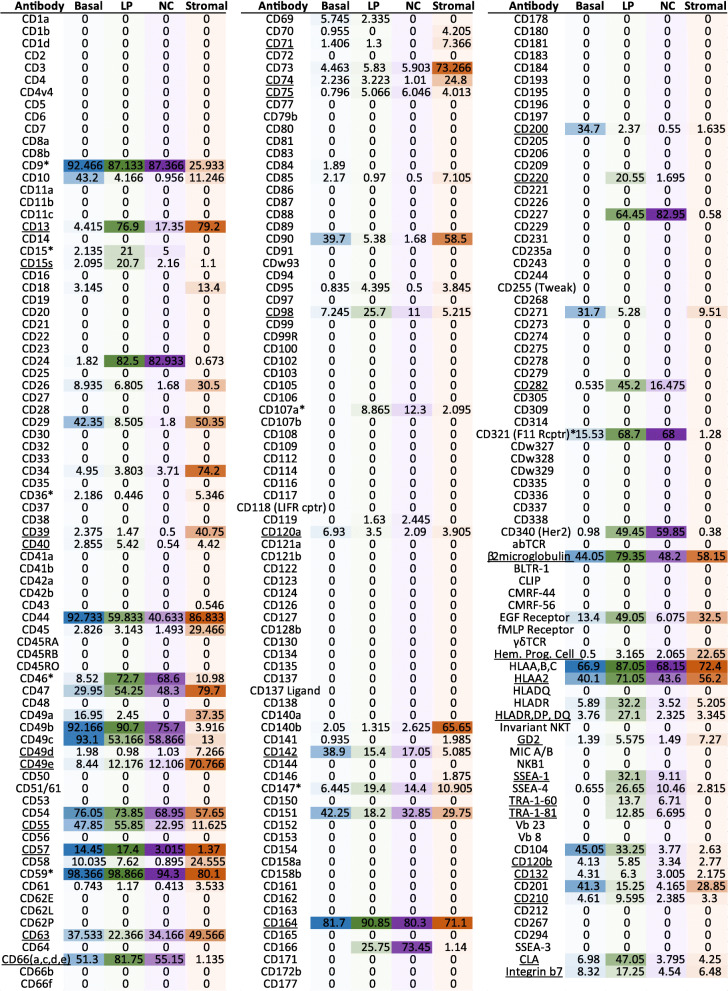
Lyoplate analysis of surface marker expression patterns in different mammary epithelial and stromal subpopulations. FACS based expression analysis of 242 surface markers using the BD Lyoplate™ Human Surface Marker Screening Panel on primary human breast cell populations (blue: basal, green: luminal progenitor (LP), purple: mature luminal (ML), orange: stromal). The values represent the mean percentage of positive cells for each surface marker antibody within two pooled donor samples from the two antibody screen replicates. Zero indicated that the percentage of cells ranged from 0 to 1% within the positive gating. CD markers underlined indicate unreported expression in breast epithelial/stromal cells. CD markers with an asterisk indicate less characterised expression in breast epithelial and stromal cells

Analysis of the screen revealed 78 out of the 242 lyoplate cell surface proteins were positive in the breast epithelial/stromal compartments (Fig. 2, Figure S2a). Without the inclusion of lineage or live/dead markers, the number of positive antibodies increased to 144 and 168, respectively (Figure S2b-c). The mean percentage of positive cells for each cell surface marker (greater than 1% positive) of the different epithelial/stromal populations was calculated (Fig. 2). As expected, our screen positively identified a number of well-known breast basal and luminal epithelial cell surface proteins including CD10, CD24, CD44, CD227, CD340 and EGFR (Fig. 2) [7, 10–12, 18, 19]. Furthermore, we identified positive expression of CD49a, CD49b, CD49c, CD47, CD54, CD73, CD90, CD95, CD151, CD271, HLA-ABC, HLA-DR, SSEA-4 and CD201 markers which were reported in primary human breast cells and tissue [20–25] and on breast organoids [26]. Surprisingly, CD117 (C-Kit), a well-known surface marker expressed on breast epithelial cells, was not detected as being positive in this screen. C-Kit [27], along with CD105 [28] were detected in breast epithelial or stromal cells, respectively, via FC. However, these studies used different clones for CD117/C-Kit and CD105 compared to the antibodies in this screen, highlighting that different antibody clones may yield contrasting results. Although the complete list of CD markers was not included in this screen, the screen contained a number of surface markers not previously examined in breast tissues. The screen identified 35 surface markers that were novel and a further 8 less characterised markers in the normal breast epithelial/stromal compartments (Fig. 2). The less characterised markers are of interest, as these markers were previously reported as having expression in normal breast tissues; however, no distinction between luminal or basal cell types was documented [29–35]. Quantification revealed several of the novel and less characterised markers were widely expressed in breast epithelial cells. For instance, CD9, CD59 and CD164 expression was detected in greater than 80% of all epithelial subpopulations (Fig. 2). Other novel markers including CD40 and CD120b were expressed in 5% or less of each epithelial subpopulation, demonstrating the heterogeneity of marker expression in the normal breast.

**Fig. 3:**
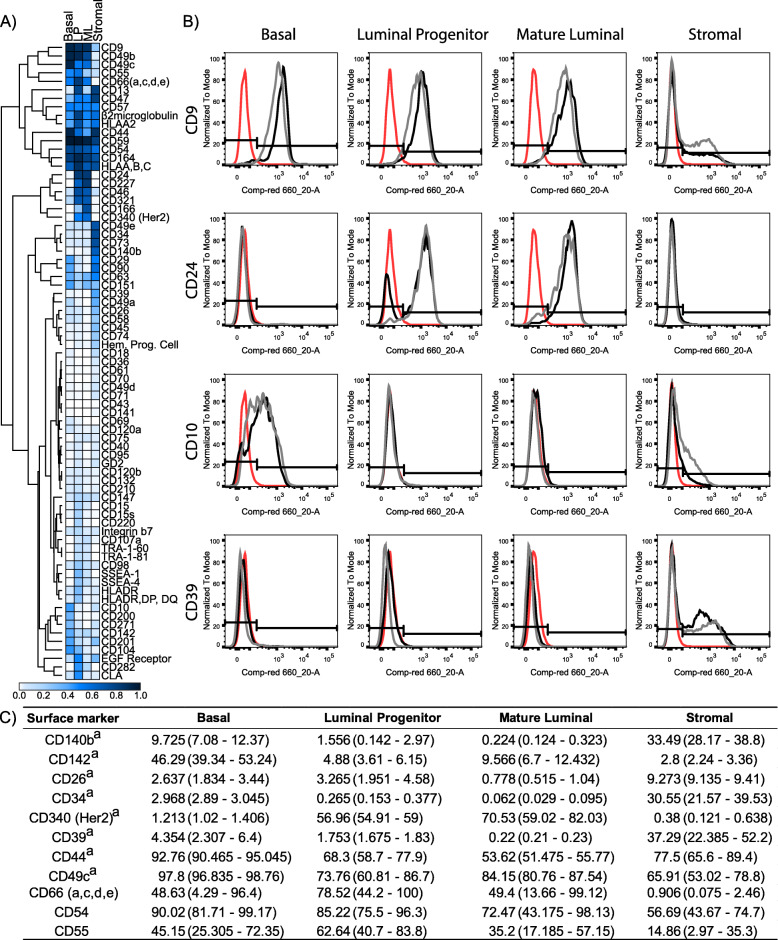
Validation of positive surface markers. **A** Heatmap showing expression of the positive detected surface markers antibodies of basal, LP, ML and stromal populations from the Lyoplate screen analysis. **B** Example histograms of show intensity staining for CD9, CD10, CD24 and CD39 compared with isotype controls (red) and the two replicate antibody screens (black and grey) in basal, LP, ML and stromal cells on a log scale. **C** 11 positive surface markers that were selected to validate the Lyoplate screen in human mammary epithelial and stromal subpopulations. Values represent the mean percentage of expression followed by the range of expression in bracket). ^a^ represent *n* = 2 independent human breast donor samples, otherwise *n* = 3

Unsupervised hierarchical clustering of the 78 positive surface markers showed several expression clusters between the different subpopulations (Fig. 3a). We observed distinct clusters exhibiting high expression in both epithelial and stromal populations (CD44, CD54, CD59, CD164, HLA A,B,C) or enriched in epithelial populations (CD9, CD49c, CD49e, CD55, and CD66(a,c,d,e)). These data indicate that these markers may contribute towards a general biological function. Other clusters of CD markers were restricted to epithelial sub-compartments including the luminal cluster encompassing of CD24, CD227, CD46, CD321, CD166 and CD340 cell surface markers, the luminal progenitor cluster (EGFR, CD282 and CLA), and the basal cell cluster (CD10, CD200, CD271, CD142, CD201 and CD104), suggesting more specialised function in these cell types. Of note, 62 of the 78 positive surface markers were expressed on stromal cells (Fig. 2), yet only a few of these markers were restricted to the stromal compartment (Fig. 3a). It is also notable that several markers were expressed in both stromal and luminal populations including CD13, CD75, CD95, CD107a, Hem. Prog. Cell and GD2 (Figs. 2 and 3a).

Marker positivity gives indication of the proportion of cells expressing these markers; it does not indicate the signal intensity or heterogenous marker expression. We generated histograms of the positive identified surface markers to determine whether heterogenous expression patterns exist in the epithelial or stromal subpopulations (Fig. 3b, Figure S3). We observed that several markers had strong signal intensities including CD9 and CD59 in all populations. Luminal cells expressing CD24, CD49b, and CD13 in the LP populations all showed strong signal intensities. Basal cells expressing CD44, CD49b and CD49c also showed strong signal intensity. However, the vast majority of markers displayed diverse fluorescence intensities, suggestive of heterogeneity marker expression. Examining the minimum and maximum signal intensities (Table 1), a small number of markers displayed a spread of signal intensities greater than 1.5 logs. Many of the markers with a broad signal intensity were only detected in less than 5% of the subpopulation. This is evident in CD36, CD39, CD73 for the basal population; CD34 for the LP population; CD29, CD34, CD39 and CD73 for the ML population, indicating that whilst these populations may have some heterogenous expression, the overall proportion of cells expressing these markers are low. The stromal population contained markers that had the most heterogenous expression, especially for cells expressing CD9, CD13, CD26, CD34, CD39, CD44, CD49a, CD54 and CD73 (Table 1, Figure S3). To validate the specificity of the screening panel, we selected well known positive markers in breast epithelial (CD44, CD340) and stromal cells (CD140b [14], CD34 [25], CD26 [28]), as well as novel/less characterised epithelial (CD142, CD49c, CD66, CD54, CD55) and novel stromal (CD39) CD markers identified from the screen (Fig. 3b) for expression analysis in an additional two independent donor samples. The resulting FC analyses indicated that all positive surface markers selected from the screen for validation were also detected in subsequent donor samples (Fig. 3c, Figure S4), however, at times the proportion of cell positivity differed. We observed antibodies such as CD140b containing 4.5-fold higher proportion in the basal compartment and a 2-fold reduction in the stromal compartment. CD142 contained a 2-3-fold reduction in luminal and stromal compartments, but a small increase in the basal compartment (Fig. 3c). Other antibodies including CD54 and CD55 showed comparable proportions between the screens and the subsequent donor samples (Fig. 3c). Whilst proportions differed in some cases between the screen and validation assays, the trend of positivity was the same, i.e. CD140b expression was most frequently detected in the stromal compartment (Fig. 2), and this trend was observed in the subsequent donor samples (Fig. 3c). This demonstrates that we have generated a robust dataset as a resource for identifying a selection of CD marker expression on normal human breast cells.

**Table 1:**
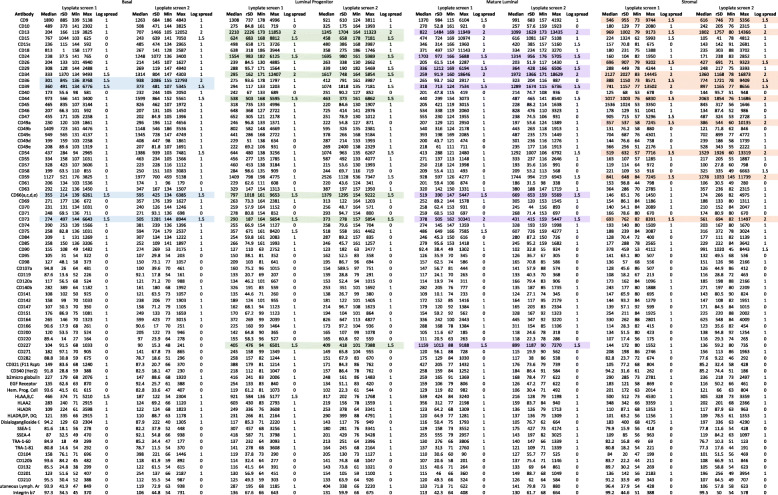
Minimum and maximum signal intensities. Cell surface markers with 1.5 log fluorescence intensities or greater are highlighted in the basal (blue), luminal progenitor (green), mature luminal (purple) and stromal (orange) compartments

**Fig. 4:**
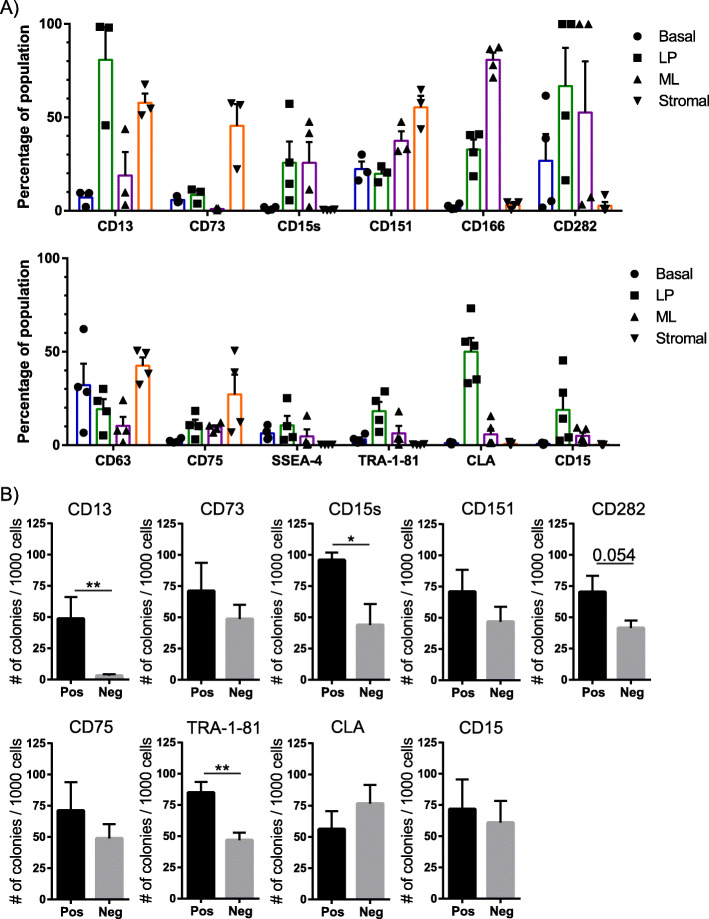
Lyoplate screen identifies novel luminal progenitor markers. **A** Analysis of variability in expression of 12 surface markers enriched in the luminal progenitor population. Bar charts show percentage of positive marker cells in each epithelial/stromal population, all error bars represent SEM. n = 3–5 independent human breast donor samples. **B** Bar chart showing the colony-forming ability of the luminal progenitor population from positive and negative surface marker expressing cells. n = 3–5 independent human breast donor samples, all error bars represent SEM. Statistical significance was calculated using two-tailed *t* test. Statistically significant differences are indicated by asterisks. * *P* < 0.05, ** *P* < 0.001

The luminal compartment is considered to be the cell of origin for most breast cancers and understanding the heterogeneity of surface marker expression in normal cells may illuminate differences in cell state with relevance to cancer initiation and progression. Focusing on the luminal compartment we investigated a selection of novel and less characterised surface markers identified from the lyoplate screen and confirmed expression in a further 3–5 donor samples (Fig. 4a). The markers were selected based on the following criteria: (i) dominant expression in the LP population (CLA, CD15s and CD15), (ii) high expression in the luminal population (CD13, CD282, TRA-1-81 and SSEA-4), (iii) moderate expression in the luminal population (CD63 and CD151) or (iv) dominant expression in the ML population (CD166). Although CD73 and CD75 markers were strongly expressed in the stromal compartment, positive cells were detected in the luminal compartments and were included for further analysis. CD166 and CD151 were also selected for further investigation. CD166 and CD151 have previously been detected in the ML and basal compartments, respectively; however, detection in the LP compartment is not well documented and warranted confirmation in a further 3–5 donor samples (Fig. 4a). FC analysis confirmed expression patterns reported in the lyoplate screen. However, we observed a range of positive cells in the luminal compartments between the different donor samples (Fig. 4a). CD13 and CD73 surface markers exhibited at least a twofold range of positive cells (Fig. 4a). CD15s, CD15, CD282 and CLA displayed a wide range of positive cells, where some donor samples exhibited a lower proportion of positive cells, between 2.5% and 33%, for these markers whilst other donors contained 50% to 100% of LP cells expressing these markers (Fig. 4a), showing the disparate variability of marker expression on human breast cells. The proportion of cells that were positive for surface markers in the ML population also varied between donor samples (Fig. 4a). Again, whilst proportions differed between the screens and the further validation assays, the trend of positivity was the same, i.e., CD13 expression was most frequently detected in the LP and stromal compartments (Figs. 2 and 4a). These data highlighted the complexities of surface marker expression and that inter-individual variation did not deviate expression patterns in the different epithelial and stromal populations.

To interrogate whether expression of surface makers enriches proliferative capacity in the LP population, purified LP cells were seeded into colony-forming assays. Surprisingly, only cells that were positive for CD13, CD15s or TRA-1-81 surface markers had increased colony-forming capacity (CFC), with the rest of the antibodies tested showing no differences in CFC between cells positive and negatively expressing these markers (Fig. 4b). Previous reports show that ALDH enriches for detection of progenitor/stem activity [3, 10, 36]. Assessing the differential expression of ALDH and the individual surface marker, we resolved the LP population into four subtypes: ALDH+surface marker Ab+, ALDH+Ab−, ALDH−Ab+, ALDH−Ab− groups (Figure S5a). Twelve surface markers were assessed for co-expression and only CD73, SSEA-4 or TRA-1-81 surface markers overlapped with ALDH expression. For instance, 6.8% of LP cells co-expressed ALDH and CD73 compared to 1.6% of LP cells that expressed CD73 only. Similar patterns were observed for the other two markers, with 7.0% of LP cells being ALDH^+^SSEA-4^+^ vs 3.6% LP cells SSEA-4^+^ and 13.2% of LP cells expressed ALDH^+^TRA-1-81^+^ vs 5.0% TRA-1-81^+^ LP cells (Figure S5a). The remaining markers were either equally distributed between ALDH positive and negative expressing cells (i.e. CD13, CD282, CD63, CD75 or CLA) or had inverse expression with ALDH expressing cells (i.e. CD15s, CD151, CD16 or CD15) (Figure S5a). Interestingly, majority of ALDH positive cells were also CD13 and CD282 positive, whilst the remaining markers only contained a smaller proportion of co-expression with ALDH positivity (Figure S5a). This result demonstrates even greater heterogeneity of the LP progenitor population beyond that can be further refined by CD markers and ALDH expression. Furthermore, colony-forming assays show that co-expression of CD73, CD282 or TRA-1-81 cell surface markers together with ALDH enriches progenitor capacity. Co-expression of ALDH and CD151 or CD15 markers increased progenitor capacity compared to cells positive for CD151 or CD15 alone (Figure S5b). An exception to this finding was CLA. CLA labelled cells had the lowest progenitor activity, suggesting CLA expression may indicate a committed LP cell subtype (Figure S5b). Showing that some of the novel/less characterised markers identified may determine different LP cell states.

## Discussion

This screen uncovered greater diversity of surface marker expression among epithelial and stromal cell lineages in normal breast tissues than what is currently reported and, whilst not a complete study of all possible cell surface markers, is a starting point for generating an overview of all surface marker expression patterns on breast epithelial and stromal cells. We identified pan-breast tissue markers such as CD9, CD54, CD59, CD164 or HLA-A,B,C that were strongly expressed in majority of epithelial and stromal breast cells, luminal lineage enriched markers including CD13, CD15, CD24, CD75, CD166, CD227, CD282 and markers that were enriched within the basal compartments, such as CD49a, CD90, CD200, CD271. This screen confirms CD expression of several well-characterised breast epithelial markers (CD10, CD24, CD44, CD227), and identified several novel surface markers including CD15s, CD75, CD164, CD282, TRA-1-81, among others.

Here we compiled a searchable dataset of surface marker expression for human breast epithelial and stromal populations that allows greater ability to refine the CDs that are functionally important for human breast development. For instance, we found that CD13 a proteolytic enzyme also known as Anpep, was strongly expressed in the LP population. *Anpep*^*-/-*^ knockout mice have delayed mammary gland development during pregnancy attributed to reduced branching morphogenesis within the duct [37]. Furthermore, transgenic mice that overexpress human ANPEP display a reciprocal phenotype including mammary glands that are hyper-branched during pregnancy [37]. The LP population is known as the secretory luminal cell type involved in alveologenesis and milk production during pregnancy. The reporting of CD13 expression in the luminal compartment, especially the LP population supports the hypothesis that CD13 positive cells may contribute to breast morphogenesis during pregnancy. This finding demonstrates the potential for our screen in identifying different cell states within the epithelial compartment, data from which can be used to explore the role of these cells within the breast tissue development.

Breast cellular heterogeneity remains a key obstacle in understanding the transition of normal cells towards cancer and how different breast cancer subtypes develop. Our screen provides a starting point for identifying novel as well as other less characterised cell surface markers that could be useful for diagnostic as well as predictive of disease progression or defining invasive tumours. The ability to identify a cell type based on marker expression/s that enables cancer development can then be used as a therapy target. CD44 has been the subject of intense breast cancer research for several decades and is considered one such example of a surface marker that is used diagnostically and for therapy. The COSMIC database reported 3.7% of breast cancers overexpress CD44 whilst 2.8% of breast cancer samples contain mutations in CD44 [38]. However, the data surrounding the role of CD44 in cancer stem cells (CSCs) or its prognostic ability can be conflicting [39]. This screen reveals that CD44 is highly expressed in all normal breast epithelial populations and corroborates previous immunostaining [5]. Therefore, it is evident that CD44 marks several cell states in normal breast and breast cancer tissues, including cells that have CSC and non-CSC roles. Our screen assessed several known CD markers and identified several novel (i.e. CD63, CD98 and CD164) and less characterised (i.e. CD46, CD107a and CD321) breast epithelial markers, of which are overexpressed in at least 5% of breast cancer samples in the COSMIC database [38]. Many studies reporting overexpression of particular markers are not always substantiated when considering the proportion of expression detected in the normal tissue. For example, CD9 is overexpressed in 10% of breast cancers [38] and has been implicated in breast tumour invasion [40], yet CD9 is expressed in approximately 90% of all normal breast epithelial cells, highlighting that CD9 may mark diverse cell functions in the different epithelial cell lineages. Use of this dataset can determine the cell types containing expression of the surface marker in the normal breast tissue and whether these CD markers are then overexpressed in cancer.

Focused investigations on a single surface marker can assist in understanding biological function of that particular marker. However, combinatorial analysis of markers will enhance our understanding of cell states in normal breast biology and tumour heterogeneity. Multiplatform single cell technologies have rapidly identified the proteomic landscape of normal breast tissue and breast cancer. However, the use of surface markers without a clear understanding of the expression pattern in breast epithelial/stromal populations may lead to interpretation difficulties of the omic data generated. Mass cytometry/imaging mass cytometry techniques have enhanced the single cell phenotypic capacity by simultaneously detecting up to triple the number of markers achieved by conventional flow cytometry. Greater proteomic and spatial architecture atlas of the breast tumour ecosystem [22, 41, 42] and normal tissue across aged breasts [43] has yielded better connections between different cell lineages. However, these datasets are limited by the availability of known surface markers for breast tissue including CD44, Her2/CD340, EGFR, CD24 markers [22, 42, 44]. A recent publication utilised several less described surface markers in relation to normal breast biology including CD47, CD54, CD73 and CD95 [26]. Using our resource, CD47 and CD54 were detected in all breast cell subpopulations at a frequency of 30-80% for each cell population, whilst CD73 and CD95 positive cells were predominately located in the LP and stromal compartments.

## Conclusions

Our resource can enhance multiplatform system such as complex surface marker staining, mass cytometry, single cell omic studies for cell lineage clarity. Using this surface marker dataset, we have identified cell lineage antibodies in addition to the standard panel of Lineage/EpCAM/CD49f which can be used to investigate the variation in epithelial and stromal compartments. These panels include (but not limited to) CD15s/CD73/CLA for further investigation into the LP compartment, whilst targeting the ML population can be carried out with the addition of CD166/CD227/CD340. Investigating heterogeneity within the basal compartment can be performed using CD29/CD142/CD271 antibodies, and antibodies targeting the stromal compartment include CD34/CD39/CD140b. These panels can be used in conventional cytometry for recoverable cellular material and further functional studies into normal breast and cancer development. Currently, mass cytometry/imaging mass cytometry datasets are limited to using known and available antibodies and many cell surface markers have not been previously reported. Multiplexing many of the surface markers that were identified in this study allows further investigation into spatial locations and relationships between different cell types in order to understand normal/disease development and functions in the breast.

## Supplementary Information

**Additional file 1: Supplemental Figure S1.** Gating strategy of antibody screen. Gating strategy to eliminate debris, doublets, dead and endothelial cells and to select the epithelial and stromal subpopulations. Percentage of positive cells for each antibody was determined based on gates drawn from the isotype control for each of the subpopulations. Gating strategy illustrated represents A) a negative and B) a positive surface marker.

**Additional file 2: Supplemental Figure S2.** Multiplexing reduces the number of positive surface marker antibodies. Pie charts depicting the proportion of positive surface marker detected in human breast single cell suspension containing A) all live cells depleted of endothelial cells, B) all live cell types and C) all cell types.

**Additional file 3: Supplemental Figure S3.** Positive surface marker expression patterns in different mammary epithelial and stromal subpopulations. Histograms show intensity staining for all positive identified antibody surface markers compared with isotype controls (red) and the duplicates of the screen in the basal, LP, ML and stromal (Black and Grey) cells on a log scale.

**Additional file 4: Supplemental Figure S4.** Validation of lyoplate screen. Representative FACS analysis depicting surface marker expression in the different epithelial/stromal subpopulations (blue: basal, green: LP, purple: ML, orange: stromal positive surface marker cells, grey illustrates negative cells).

**Additional file 5: Supplemental Figure S5.** Luminal progenitor activity in surface marker and ALDH expression. A) Analysis of variability in expression of ALDH and the 12 surface markers in the luminal progenitor populations. Bar charts show percentage of positive marker cells in each of the LP subpopulations, all error bars represent SEM. n=3-5 independent human breast donor samples. B) Stacked bar chart showing the colony forming ability of the luminal progenitor ALDH-Ab+/ALDH+Ab+ or ALDH+Ab-/ALDH-Ab- subpopulations. n=3-5 independent human breast donor samples, error bars represent SEM. Statistical significance was calculated using an ANOVA and Tukey’s multiple comparison test. Statistical significance differences are indicated by asterisks * *p*< 0.05 and ** *p*< 0.01.

**Additional file 6.**

## Data Availability

The dataset generated, used and analysed in this study available from the corresponding author upon request.

## Abbreviations

ALDH: Aldehyde dehydrogenase
CD: Cluster of differentiation
CFC: Colony-forming capacity
FC: Flow cytometry
K: Keratin
LP: Luminal progenitor
ML: Mature luminal
TDLU: Terminal ductal lobular unit

## References

[1] Petersen OW, Høyer PE, van Deurs B (1987). Frequency and distribution of estrogen receptor-positive cells in normal, nonlactating human breast tissue. Cancer Res..

[2] Sleeman KE, Kendrick H, Robertson D, Isacke CM, Ashworth A, Smalley MJ (2007). Dissociation of estrogen receptor expression and in vivo stem cell activity in the mammary gland. J Cell Biol..

[3] Shehata M, Teschendorff A, Sharp G, Novcic N, Russell IA, Avril S, Prater M, Eirew P, Caldas C, Watson CJ, Stingl J (2012). Phenotypic and functional characterisation of the luminal cell hierarchy of the mammary gland. Breast Cancer Res..

[4] Wellings SR, Jensen HM, Marcum RG (1975). An atlas of subgross pathology of the human breast with special reference to possible precancerous lesions. J Natl Cancer Inst..

[5] Santagata S, Thakkar A, Ergonul A, Wang B, Woo T, Hu R, Harrell JC, McNamara G, Schwede M, Culhane AC, Kindelberger D, Rodig S, Richardson A, Schnitt SJ, Tamimi RM, Ince TA (2014). Taxonomy of breast cancer based on normal cell phenotype predicts outcome. J Clin Invest..

[6] Gusterson BA, Ross DT, Heath VJ, Stein T. Basal cytokeratins and their relationship to the cellular origin and functional classification of breast cancer. Breast Cancer Res. 2005;7(4):143–8. 10.1186/bcr1041. 10.1186/bcr1041 PMC1175069 15987465

[7] Stingl J, Eaves CJ, Kuusk U, Emerman JT (1998). Phenotypic and functional characterization in vitro of a multipotent epithelial cell present in the normal adult human breast. Differentiation..

[8] Stingl J, Eaves CJ, Zandieh I, Emerman JT (2001). Characterization of bipotent mammary epithelial progenitor cells in normal adult human breast tissue. Breast Cancer Res Treat..

[9] Gudjonsson T, Villadsen R, Nielsen HL, Rønnov-Jessen L, Bissell MJ, Petersen OW (2002). Isolation, immortalization, and characterization of a human breast epithelial cell line with stem cell properties. Genes Dev..

[10] Ginestier C, Hur MH, Charafe-Jauffret E, Monville F, Dutcher J, Brown M, Jacquemier J, Viens P, Kleer CG, Liu S, Schott A, Hayes D, Birnbaum D, Wicha MS, Dontu G (2007). ALDH1 Is a Marker of Normal and Malignant Human Mammary Stem Cells and a Predictor of Poor Clinical Outcome. Cell Stem Cell..

[11] Garbe JC, Pepin F, Pelissier FA, Sputova K, Fridriksdottir AJ, Guo DE, Villadsen R, Park M, Petersen OW, Borowsky AD, Stampfer MR, LaBarge MA (2012). Accumulation of multipotent progenitors with a basal differentiation bias during aging of human mammary epithelia. Cancer Res..

[12] Keller PJ, Lin AF, Arendt LM, Klebba I, Jones AD, Rudnick JA, DiMeo TA, Gilmore H, Jefferson DM, Graham RA, Naber SP, Schnitt S, Kuperwasser C (2010). Mapping the cellular and molecular heterogeneity of normal and malignant breast tissues and cultured cell lines. Breast Cancer Res..

[13] Villadsen R, Fridriksdottir AJ, Rønnov-Jessen L, Gudjonsson T, Rank F, LaBarge MA (2007). Evidence for a stem cell hierarchy in the adult human breast. J Cell Biol..

[14] Lim E, Vaillant F, Wu D, Forrest NC, Pal B, Hart AH (2009). Aberrant luminal progenitors as the candidate target population for basal tumor development in BRCA1 mutation carriers. Nat Med..

[15] Nguyen QH, Pervolarakis N, Blake K, Ma D, Davis RT, James N (2018). Profiling human breast epithelial cells using single cell RNA sequencing identifies cell diversity. Nat Commun..

[16] Lawson DA, Bhakta NR, Kessenbrock K, Prummel KD, Yu Y, Takai K, Zhou A, Eyob H, Balakrishnan S, Wang CY, Yaswen P, Goga A, Werb Z (2015). Single-cell analysis reveals a stem-cell program in human metastatic breast cancer cells. Nature..

[17] Shehata M, Stingl J. Purification of distinct subsets of epithelial cells from normal human breast tissue. Methods Mol Biol. 2017;1501:261–76. 10.1007/978-1-4939-6475-8_13. 10.1007/978-1-4939-6475-8_13 27796958

[18] Stingl J, Raouf A, Emerman JT, Eaves CJ (2005). Epithelial Progenitors in the Normal Human Mammary Gland. J Mammary Gland Biol Neoplasia..

[19] Keller PJ, Arendt LM, Skibinski A, Logvinenko T, Klebba I, Dong S, Smith AE, Prat A, Perou CM, Gilmore H, Schnitt S, Naber SP, Garlick JA, Kuperwasser C (2012). Defining the cellular precursors to human breast cancer. PNAS..

[20] Suzuki RN, Entwistle A, Atherton AJ, Clarke C, Lakhani SR, O’Hare MJ (2002). The expression patterns of integrin subunits on human breast tissues obtained during pregnancy. Cell Biol Int..

[21] Yang XH, Richardson AL, Torres-Arzayus MI, Zhou P, Sharma C, Kazarov AR, Andzelm MM, Strominger JL, Brown M, Hemler ME (2008). CD151 accelerates breast cancer by regulating α6 integrin function, signaling, and molecular organization. Cancer Res..

[22] Wagner J, Rapsomaniki MA, Chevrier S, Anzeneder T, Langwieder C, Dykgers A (2019). A Single-Cell Atlas of the Tumor and Immune Ecosystem of Human Breast Cancer. Cell.

[23] Kumar B, Prasad M, Bhat-Nakshatri P, Anjanappa M, Kalra M, Marino N, Storniolo AM, Rao X, Liu S, Wan J, Liu Y, Nakshatri H (2018). Normal breast-derived epithelial cells with luminal and intrinsic subtype-enriched gene expression document interindividual differences in their differentiation Cascade. Cancer Res..

[24] Nakshatri H, Anjanappa M, Bhat-Nakshatri P (2015). Ethnicity-dependent and -independent heterogeneity in healthy normal breast hierarchy impacts tumor characterization. Sci Rep..

[25] Fridriksdottir AJ, Villadsen R, Morsing M, Klitgaard MC, Kim J, Petersen OW, Rønnov-Jessen L (2017). Proof of region-specific multipotent progenitors in human breast epithelia. PNAS..

[26] Rosenbluth JM, Schackmann RCJ, Gray GK, Selfors LM, Li CM-C, Boedicker M, Kuiken HJ, Richardson A, Brock J, Garber J, Dillon D, Sachs N, Clevers H, Brugge JS (2020). Organoid cultures from normal and cancer-prone human breast tissues preserve complex epithelial lineages. Nat Commun..

[27] Fridriksdottir AJ, Kim J, Villadsen R, Klitgaard MC, Hopkinson BM, Petersen OW, Rønnov-Jessen L (2015). Propagation of oestrogen receptor-positive and oestrogen-responsive normal human breast cells in culture. Nat Commun..

[28] Morsing M, Klitgaard MC, Jafari A, Villadsen R, Kassem M, Petersen OW, Rønnov-Jessen L (2016). Evidence of two distinct functionally specialized fibroblast lineages in breast stroma. Breast Cancer Res..

[29] Jamil F, Peston D, Shousha S (2001). CD9 immunohistochemical staining of breast carcinoma: Unlikely to provide useful prognostic information for routine use. Histopathology..

[30] Croce MV (2007). Lewis x is Highly Expressed in Normal Tissues: a Comparative Immunohistochemical Study and Literature Revision. Pathol. Oncol. Res..

[31] Defilippis RA, Chang H, Dumont N, Rabban JT, Chen Y-Y, Fontenay GV (2012). CD36 Repression Activates a Multicellular Stromal Program Shared by High Mammographic Density and Tumor Tissues.

[32] Thorsteinsson L, O’Dowd GM, Harrington PM, Johnson PM (1998). The complement regulatory proteins CD46 and CD59, but not CD55, are highly expressed by glandular epithelium of human breast and colorectal tumour tissues. APMIS..

[33] Wang Q, Yao J, Jin Q, Wang X, Zhu H, Huang F, Wang W, Qiang J, Ni Q (2017). LAMP1 expression is associated withpoor prognosis in breast cancer. Oncol Lett..

[34] Pinheiro C, Albergaria A, Paredes J, Sousa B, Dufloth R, Vieira D, Schmitt F, Baltazar F (2010). Monocarboxylate transporter 1 is up-regulated in basal-like breast carcinoma. Histopathology..

[35] Naik MU, Naik TU, Suckow AT, Duncan MK, Naik UP (2008). Attenuation of junctional adhesion molecule-A is a contributing factor for breast cancer cell invasion. Cancer Res..

[36] Eirew P, Kannan N, Knapp DJHF, Vaillant F, Emerman JT, Lindeman GJ, Visvader JE, Eaves CJ (2012). Aldehyde Dehydrogenase Activity Is a Biomarker of Primitive Normal Human Mammary Luminal Cells. Stem Cells..

[37] Kolb AF, Sorrell D, Lassnig C, Lillico S, Carlisle A, Neil C, Robinson C, Müller M, Whitelaw CBA (2013). Mammary gland development is delayed in mice deficient for aminopeptidase N. Transgenic Res..

[38] Tate JG, Bamford S, Jubb HC, Sondka Z, Beare DM, Bindal N, Boutselakis H, Cole CG, Creatore C, Dawson E, Fish P, Harsha B, Hathaway C, Jupe SC, Kok CY, Noble K, Ponting L, Ramshaw CC, Rye CE, Speedy HE, Stefancsik R, Thompson SL, Wang S, Ward S, Campbell PJ, Forbes SA (2019). COSMIC: The Catalogue Of Somatic Mutations In Cancer. Nucleic Acids Res..

[39] Louderbough JMV, Schroeder JA. Understanding the dual nature of CD44 in breast cancer progression. Mol. Cancer Res. 2011;9(12):1573–86. 10.1158/1541-7786.MCR-11-0156. 10.1158/1541-7786.MCR-11-0156 21970856

[40] Rappa G, Green TM, Karbanová J, Corbeil D, Lorico A (2015). Tetraspanin CD9 determines invasiveness and tumorigenicity of human breast cancer cells. Oncotarget..

[41] Ali HR, Jackson HW, Zanotelli VRT, Danenberg E, Fischer JR, Bardwell H (2020). Imaging mass cytometry and multiplatform genomics define the phenogenomic landscape of breast cancer. Nat Cancer..

[42] Jackson HW, Fischer JR, Zanotelli VRT, Ali HR, Mechera R, Soysal SD, Moch H, Muenst S, Varga Z, Weber WP, Bodenmiller B (2020). The single-cell pathology landscape of breast cancer. Nature..

[43] Pelissier Vatter FA, Schapiro D, Chang H, Borowsky AD, Lee JK, Parvin B, Stampfer MR, LaBarge MA, Bodenmiller B, Lorens JB (2018). High-Dimensional Phenotyping Identifies Age-Emergent Cells in Human Mammary Epithelia. Cell Rep..

[44] Giesen C, Wang HAO, Schapiro D, Zivanovic N, Jacobs A, Hattendorf B, Schüffler PJ, Grolimund D, Buhmann JM, Brandt S, Varga Z, Wild PJ, Günther D, Bodenmiller B (2014). Highly multiplexed imaging of tumor tissues with subcellular resolution by mass cytometry. Nat Methods..

