# Supplementary figures and images for: High-throughput surface marker screen on primary human breast tissues reveals further cellular heterogeneity
Source: Breast Cancer Res. 2021 Jun 13;23:66. doi: 10.1186/s13058-021-01444-5 (PMC8201685; doi:10.1186/s13058-021-01444-5)

A) eg of negative ab

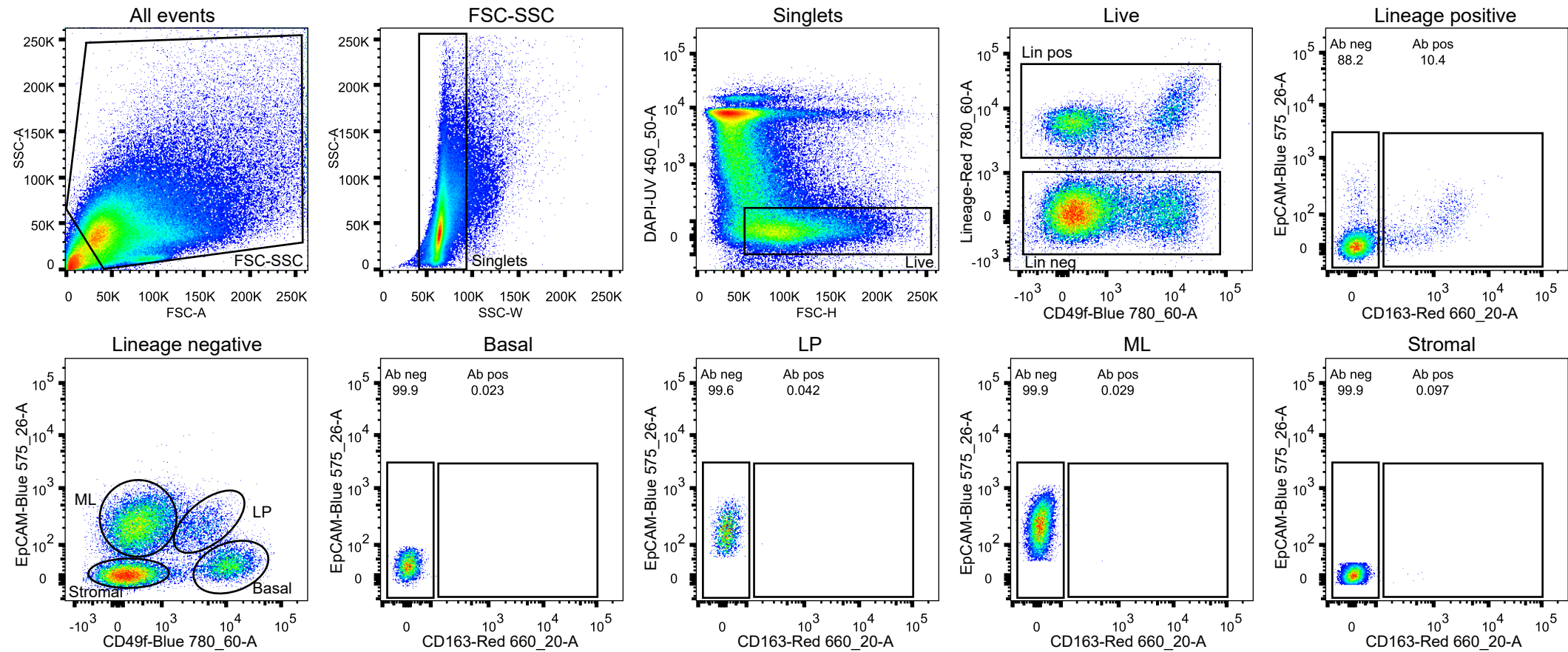

Supplement: Supplementary file 1 — Additional file 1: Supplemental Figure S1. Gating strategy of antibody screen. Gating strategy to eliminate debris, doublets, dead and endothelial cells and to select the epithelial and stromal subpopulations. Percentage of positive cells for each antibody was determined based on gates drawn from the isotype control for each of the subpopulations. Gating strategy illustrated represents A) a negative and B) a positive surface marker. [file 13058_2021_1444_MOESM1_ESM.zip › Virtanen_Additional_file_1a.pdf]

B) eg of positive ab

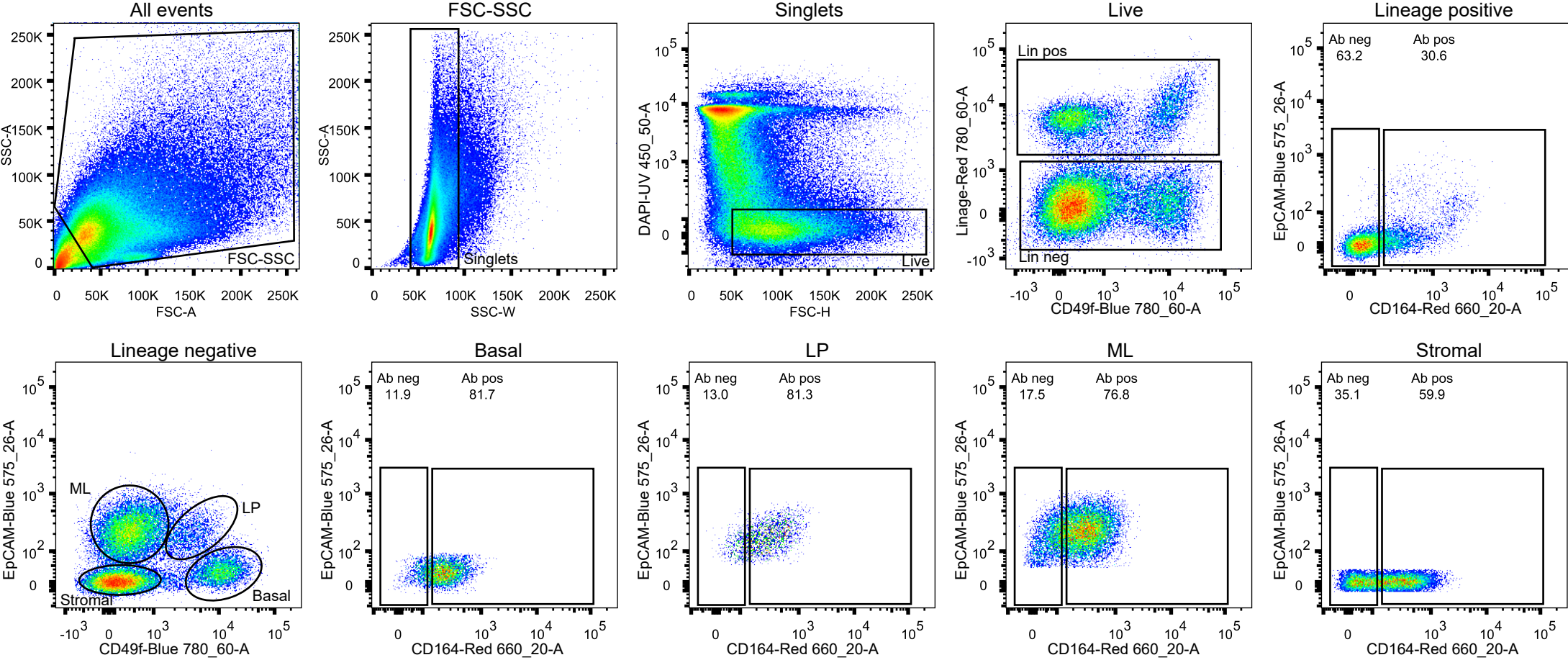

Supplement: Supplementary file 1 — Additional file 1: Supplemental Figure S1. Gating strategy of antibody screen. Gating strategy to eliminate debris, doublets, dead and endothelial cells and to select the epithelial and stromal subpopulations. Percentage of positive cells for each antibody was determined based on gates drawn from the isotype control for each of the subpopulations. Gating strategy illustrated represents A) a negative and B) a positive surface marker. [file 13058_2021_1444_MOESM1_ESM.zip › Virtanen_Additional_file_1b.pdf]

Luminal

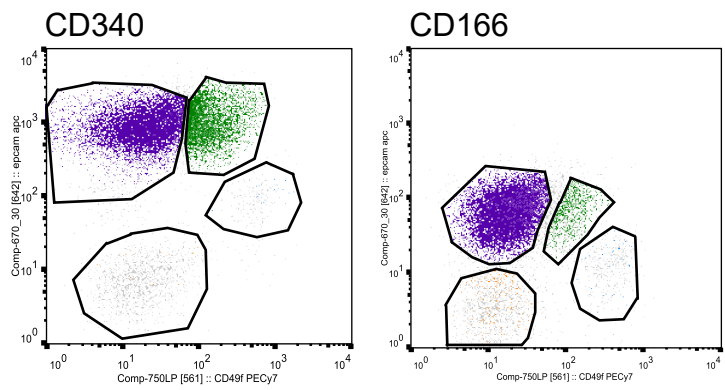

Epithelial

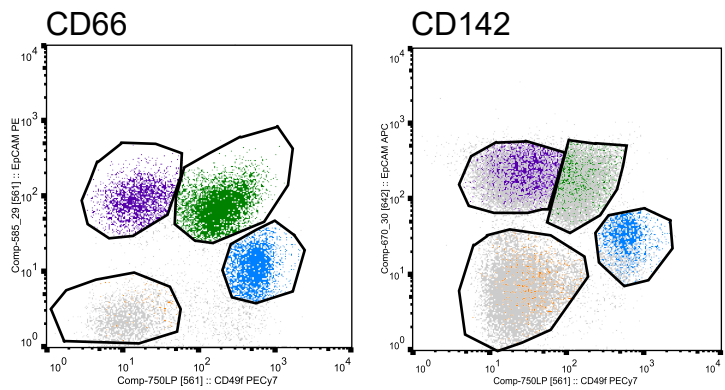

All pops

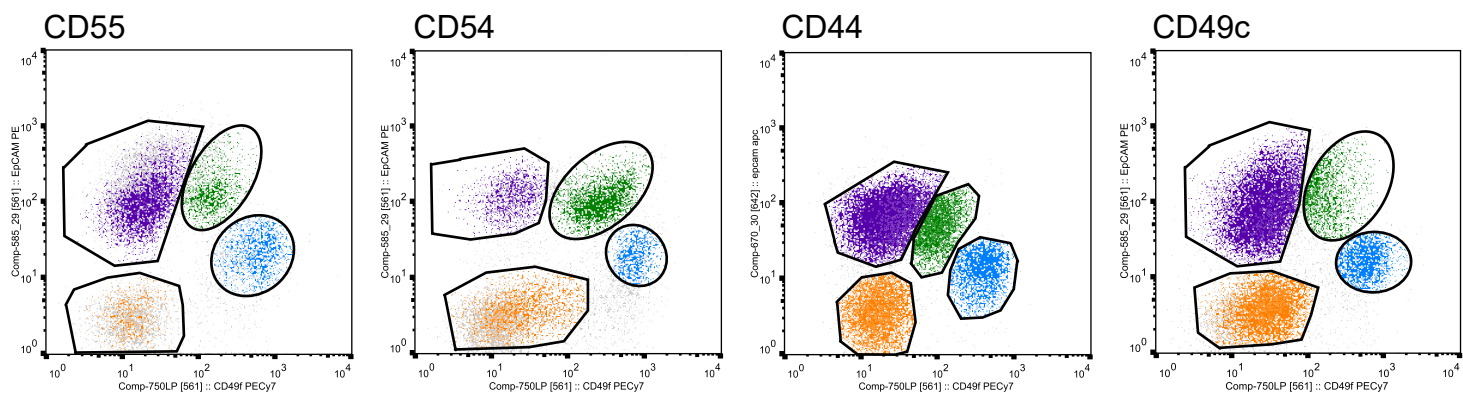

Stromal

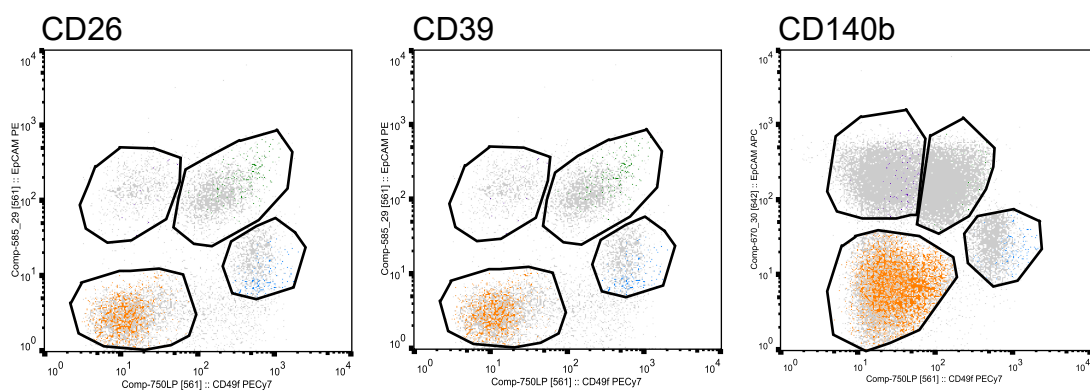

Supplement: Supplementary file 4 — Additional file 4: Supplemental Figure S4. Validation of lyoplate screen. Representative FACS analysis depicting surface marker expression in the different epithelial/stromal subpopulations (blue: basal, green: LP, purple: ML, orange: stromal positive surface marker cells, grey illustrates negative cells). [file 13058_2021_1444_MOESM4_ESM.pdf]

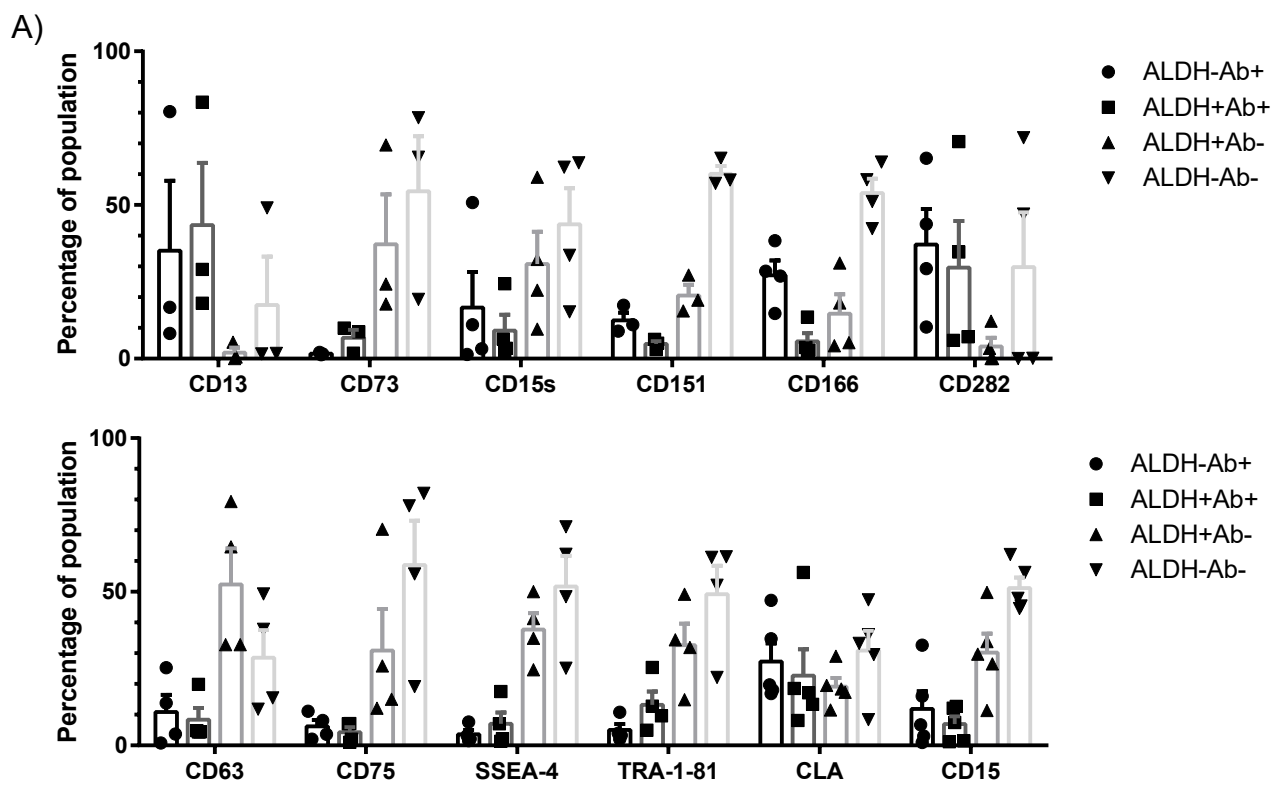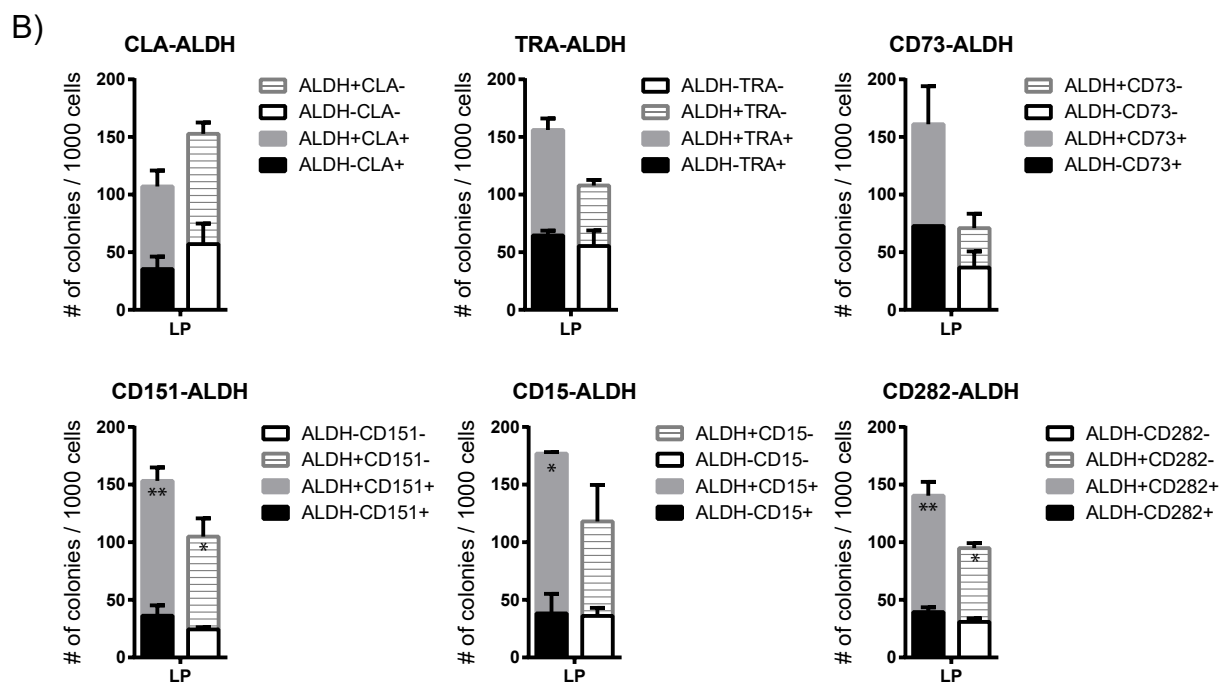

Supplement: Supplementary file 5 — Additional file 5: Supplemental Figure S5. Luminal progenitor activity in surface marker and ALDH expression. A) Analysis of variability in expression of ALDH and the 12 surface markers in the luminal progenitor populations. Bar charts show percentage of positive marker cells in each of the LP subpopulations, all error bars represent SEM. n=3-5 independent human breast donor samples. B) Stacked bar chart showing the colony forming ability of the luminal progenitor ALDH-Ab+/ALDH+Ab+ or ALDH+Ab-/ALDH-Ab- subpopulations. n=3-5 independent human breast donor samples, error bars represent SEM. Statistical significance was calculated using an ANOVA and Tukey’s multiple comparison test. Statistical significance differences are indicated by asterisks * p< 0.05 and ** p< 0.01. [file 13058_2021_1444_MOESM5_ESM.pdf]
