# Supplementary material for: High-throughput surface marker screen on primary human breast tissues reveals further cellular heterogeneity
Source: Breast Cancer Res. 2021 Jun 13;23:66. doi: 10.1186/s13058-021-01444-5 (PMC8201685; doi:10.1186/s13058-021-01444-5)

A) **Live-Lin neg**

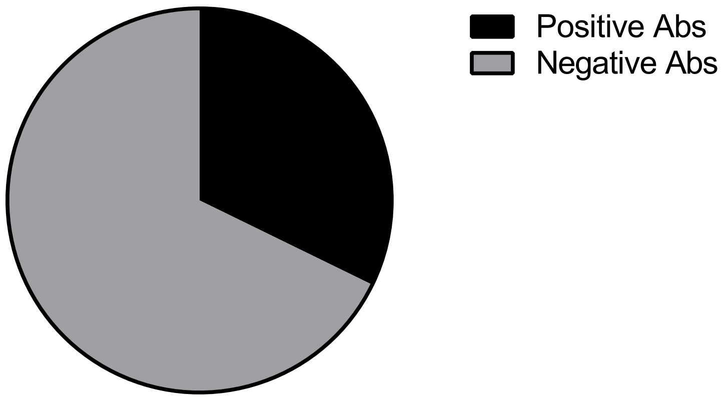

B) **DAPI negative cells**

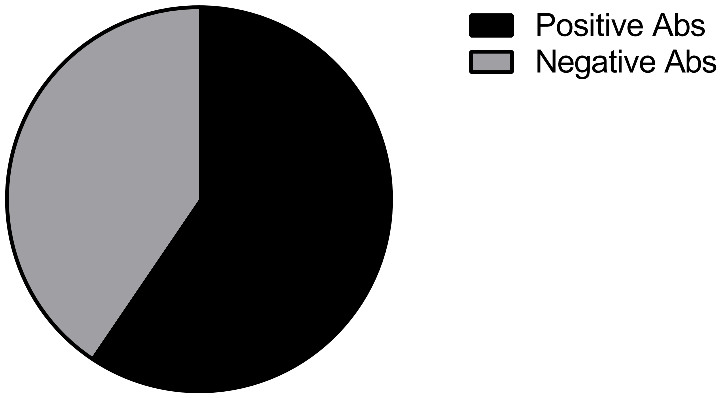

C) **All events**

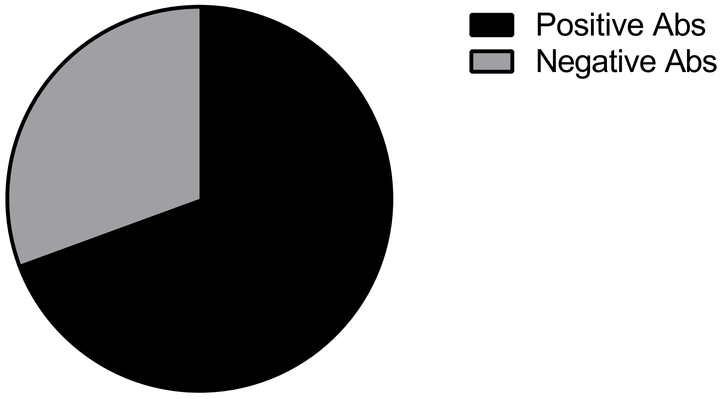

Supplement: Supplementary file 2 — Additional file 2: Supplemental Figure S2. Multiplexing reduces the number of positive surface marker antibodies. Pie charts depicting the proportion of positive surface marker detected in human breast single cell suspension containing A) all live cells depleted of endothelial cells, B) all live cell types and C) all cell types. [file 13058_2021_1444_MOESM2_ESM.pdf]
