# Supplementary material for: High-throughput surface marker screen on primary human breast tissues reveals further cellular heterogeneity
Source: Breast Cancer Res. 2021 Jun 13;23:66. doi: 10.1186/s13058-021-01444-5 (PMC8201685; doi:10.1186/s13058-021-01444-5)
Supplement: Supplementary file 3 — Additional file 3: Supplemental Figure S3. Positive surface marker expression patterns in different mammary epithelial and stromal subpopulations. Histograms show intensity staining for all positive identified antibody surface markers compared with isotype controls (red) and the duplicates of the screen in the basal, LP, ML and stromal (Black and Grey) cells on a log scale. [file 13058_2021_1444_MOESM3_ESM.pdf]

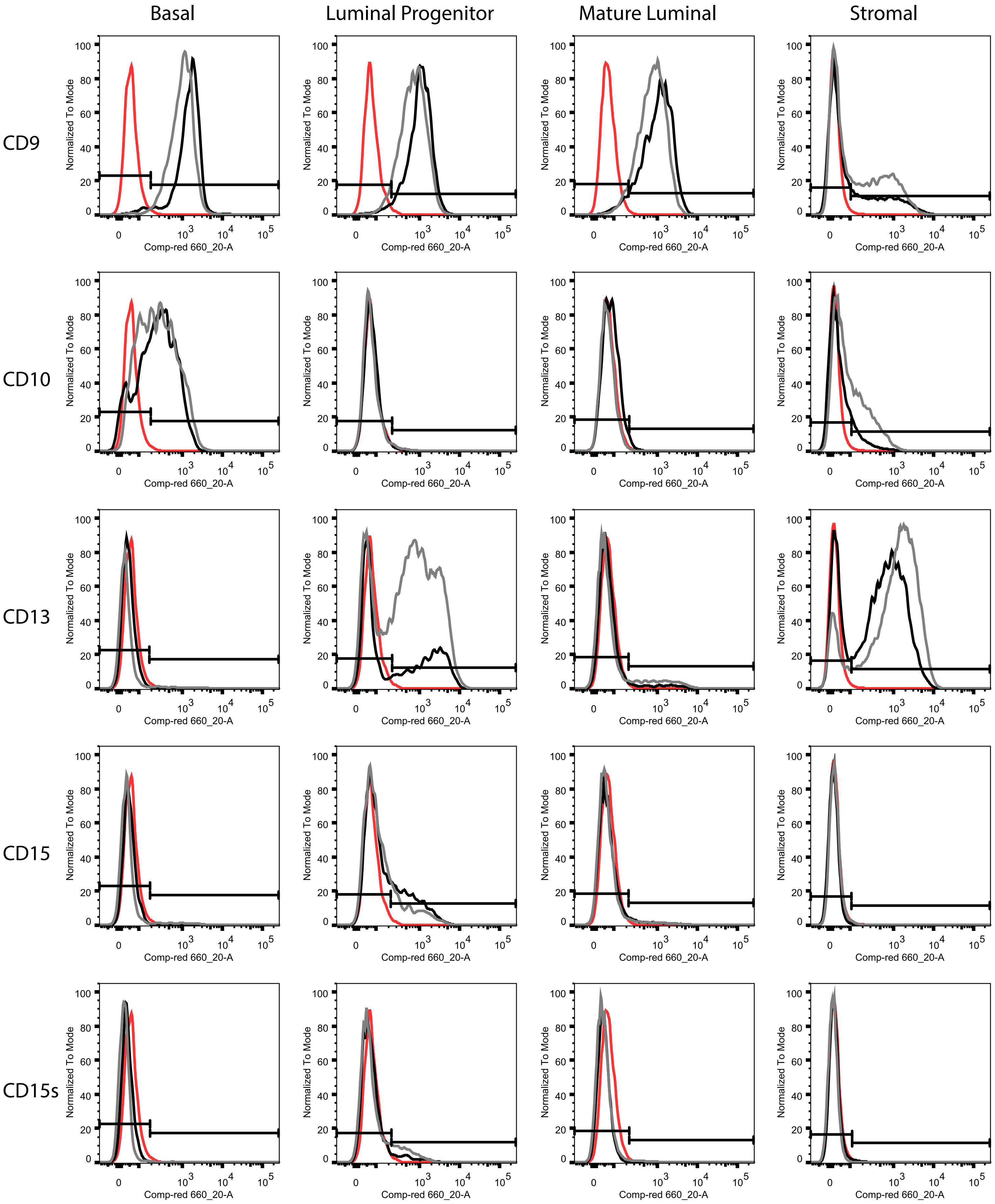
Basal
Luminal Progenitor
Mature Luminal
Stromal
CD9
CD10
CD13
CD15
CD15s
Normalized To Mode
Comp-red 660_20-A
0
10^3
10^4
10^5
0
20
40
60
80
100

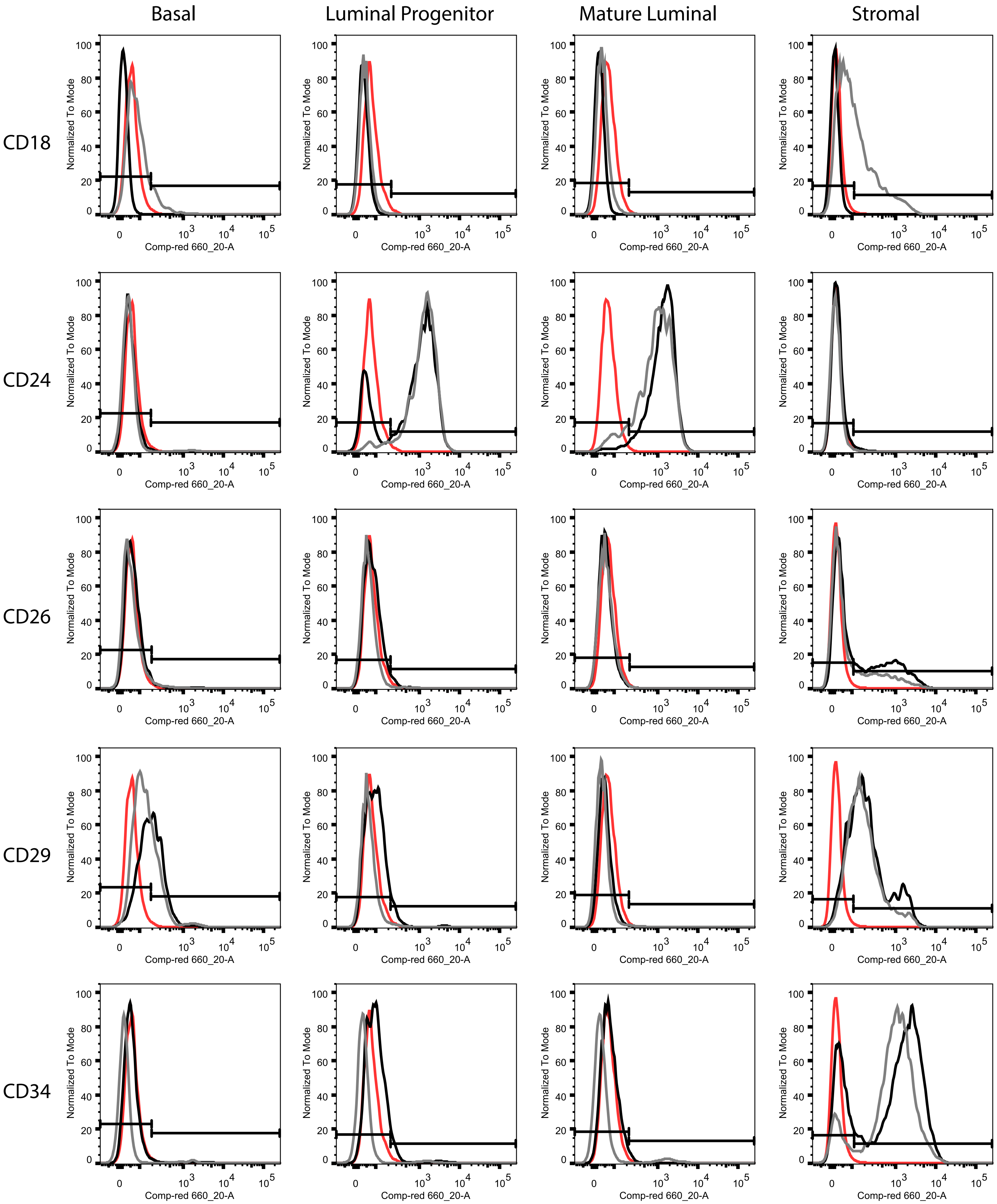
Basal
Luminal Progenitor
Mature Luminal
Stromal
CD18
CD24
CD26
CD29
CD34
Normalized To Mode
Comp-red 660_20-A
0
10^3
10^4
10^5
0
20
40
60
80
100

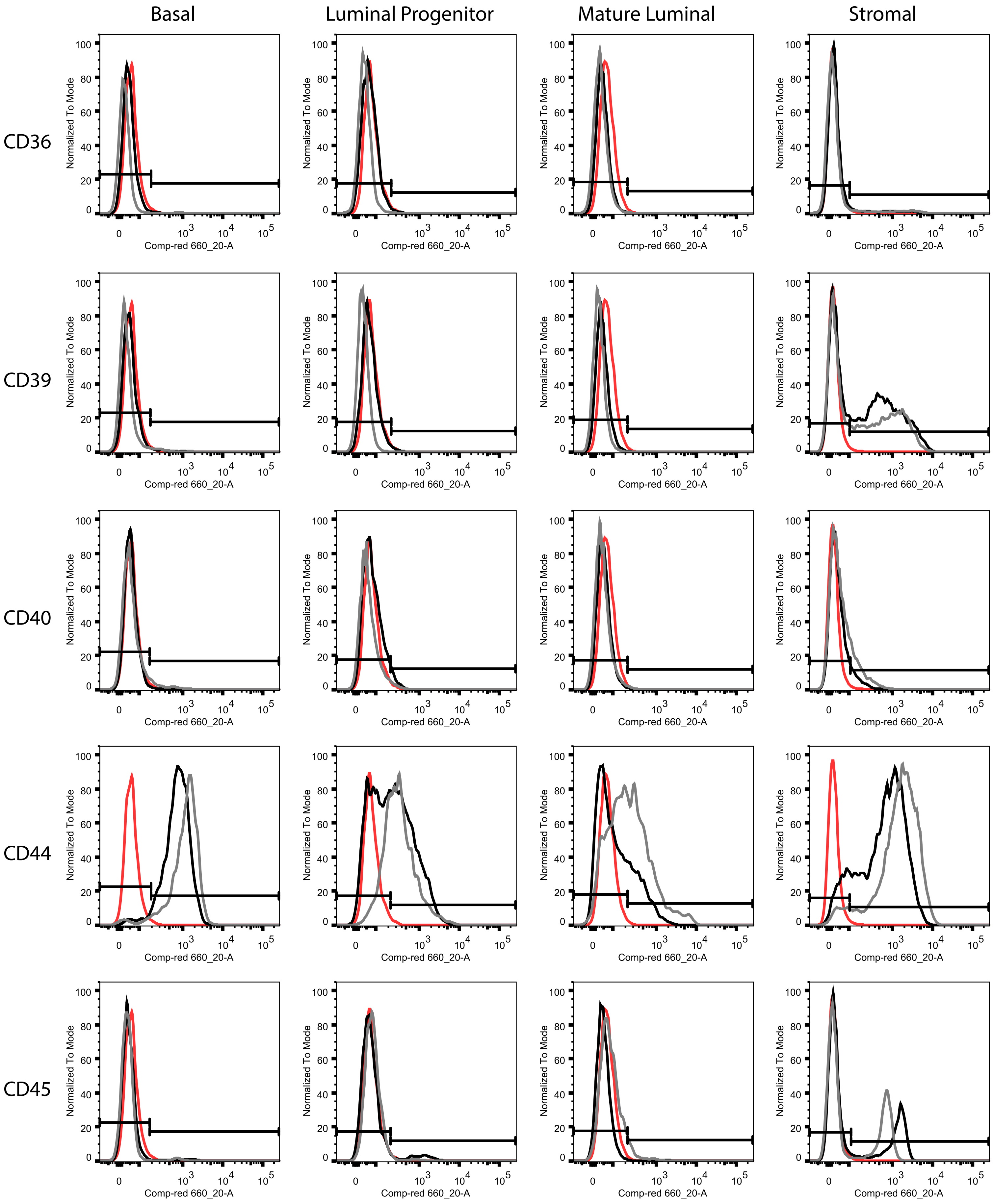

Basal
Luminal Progenitor
Mature Luminal
Stromal
CD36
CD39
CD40
CD44
CD45
Normalized To Mode
Comp-red 660_20-A
0
20
40
60
80
100
10^3
10^4
10^5

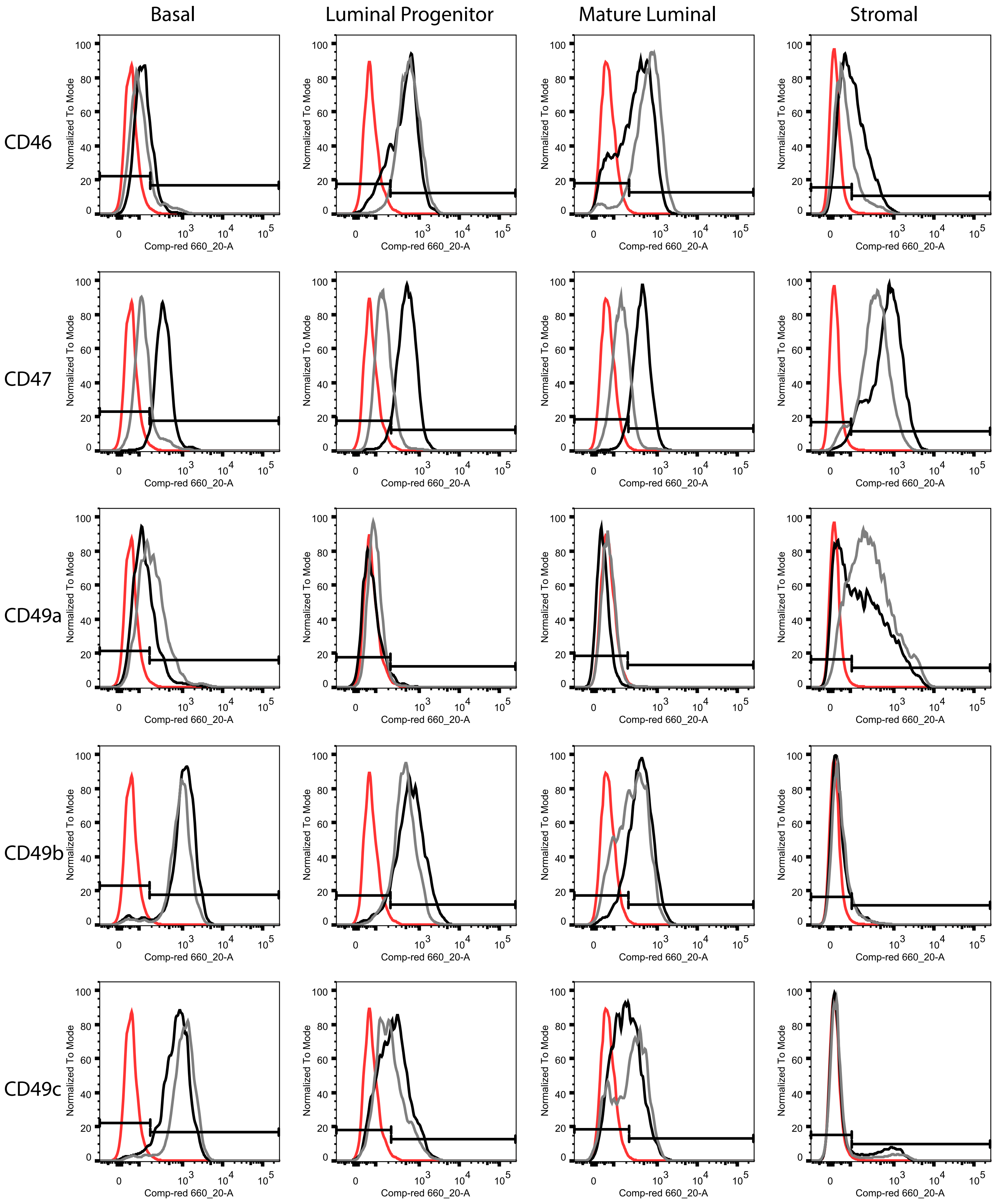
Basal
Luminal Progenitor
Mature Luminal
Stromal
CD46
CD47
CD49a
CD49b
CD49c
Normalized To Mode
100
80
60
40
20
0
0
10^3
10^4
10^5
Comp-red 660_20-A

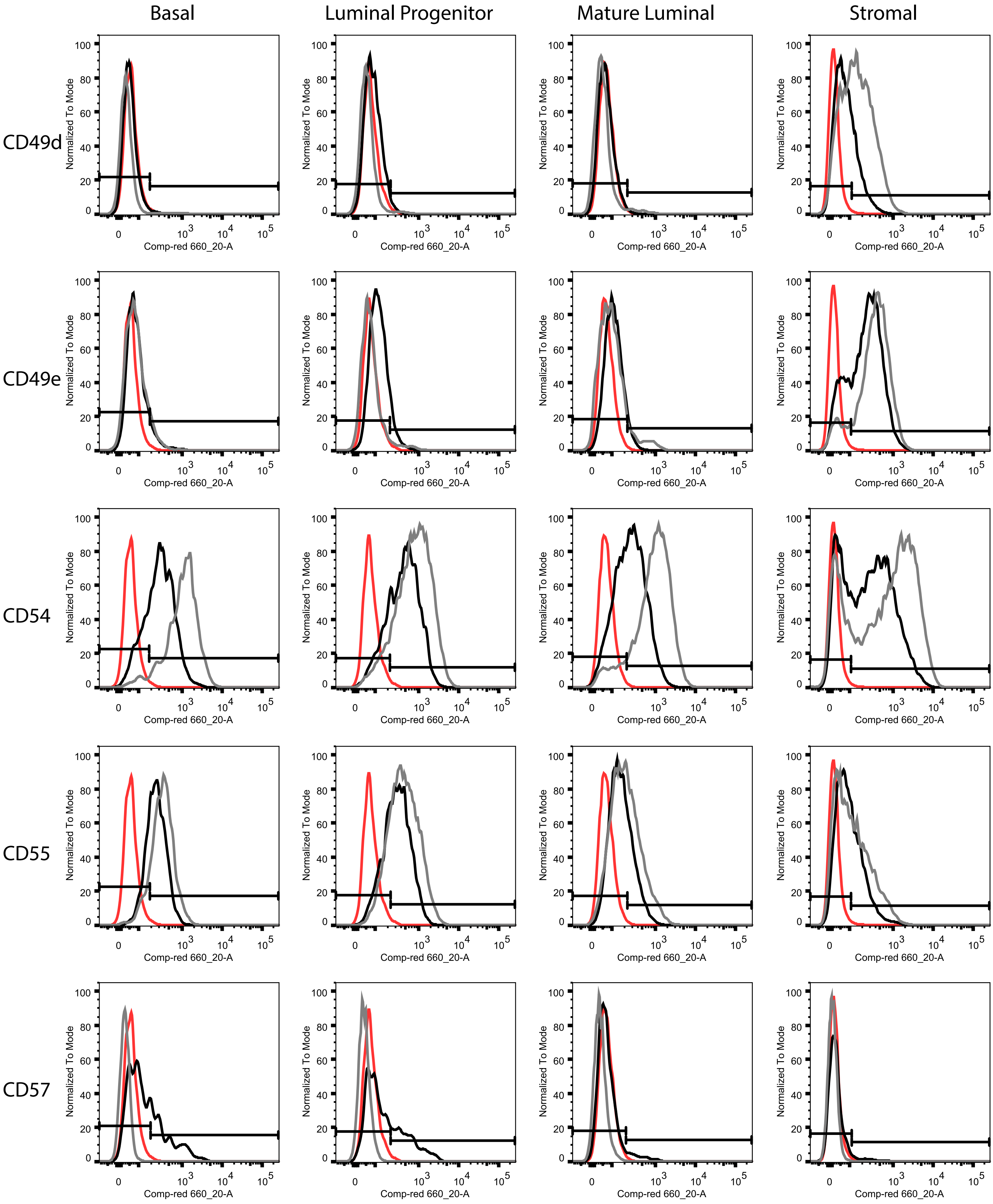

Basal
Luminal Progenitor
Mature Luminal
Stromal
CD49d
CD49e
CD54
CD55
CD57
Normalized To Mode
Comp-red 660_20-A

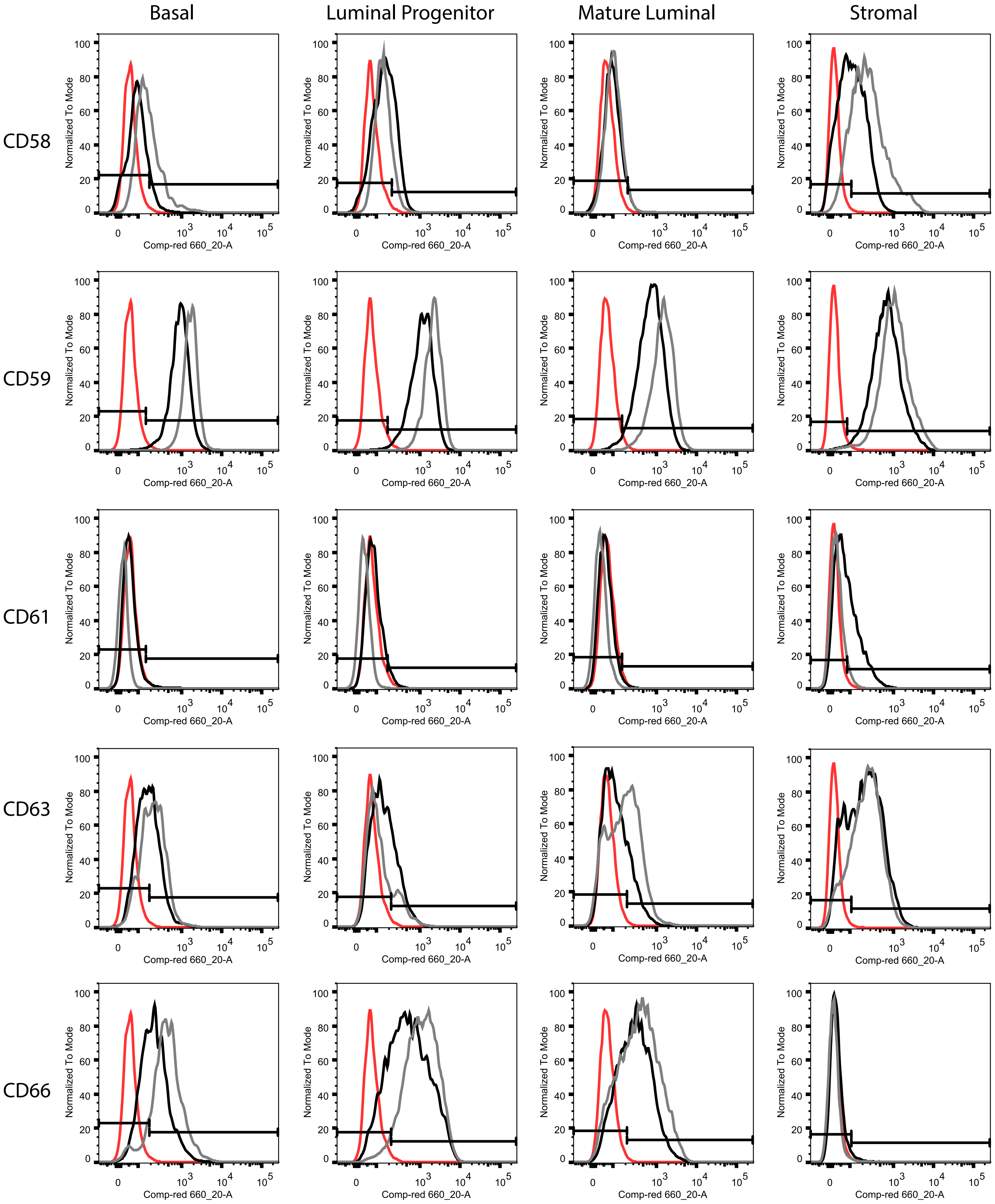

Basal
Luminal Progenitor
Mature Luminal
Stromal
CD58
CD59
CD61
CD63
CD66
Normalized To Mode
Comp-red 660_20-A

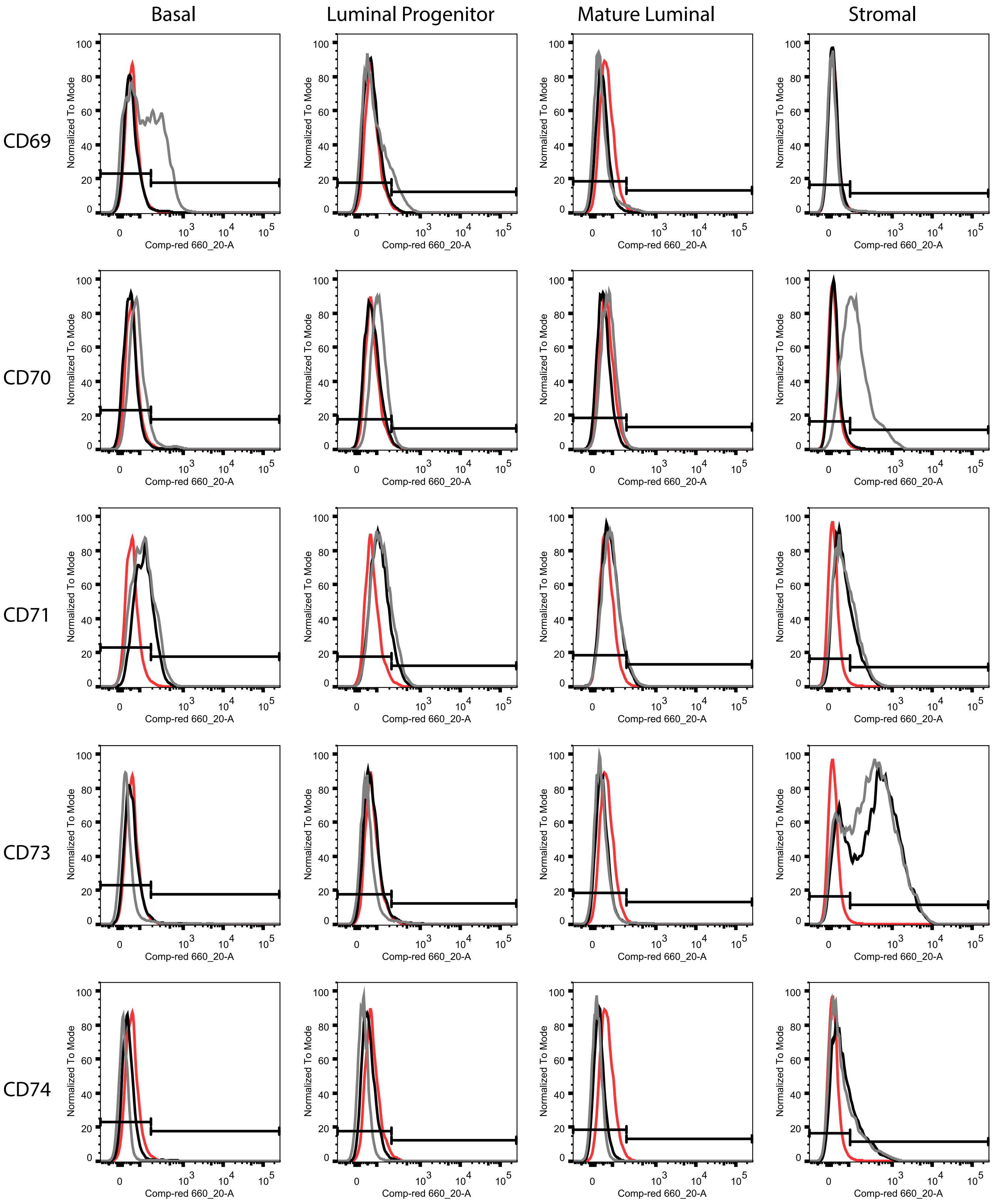
Basal
Luminal Progenitor
Mature Luminal
Stromal
CD69
CD70
CD71
CD73
CD74
Normalized To Mode
100
80
60
40
20
0
0
10^3
10^4
10^5
Comp-red 660_20-A

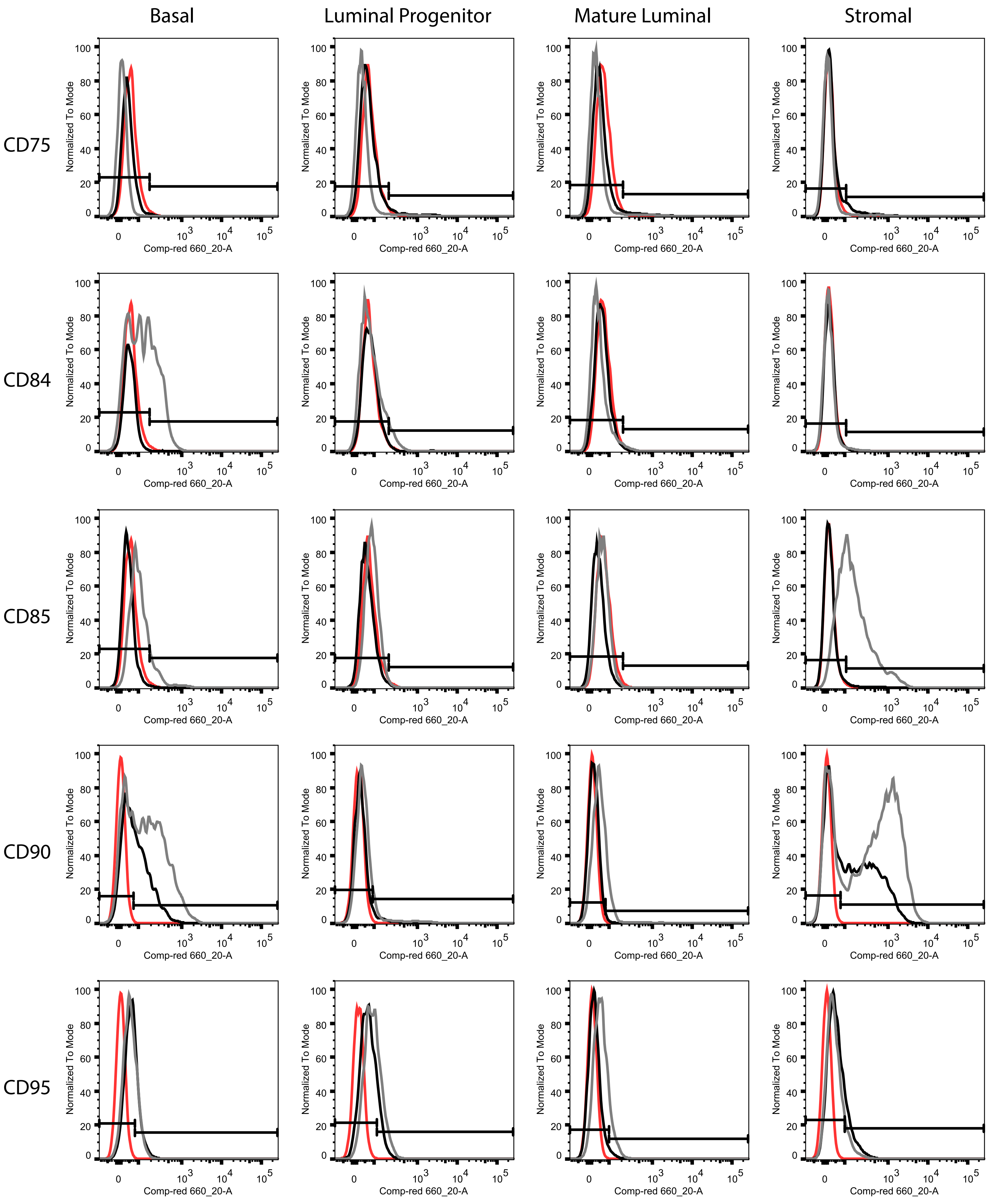

Basal
Luminal Progenitor
Mature Luminal
Stromal
CD75
CD84
CD85
CD90
CD95
Normalized To Mode
Comp-red 660_20-A

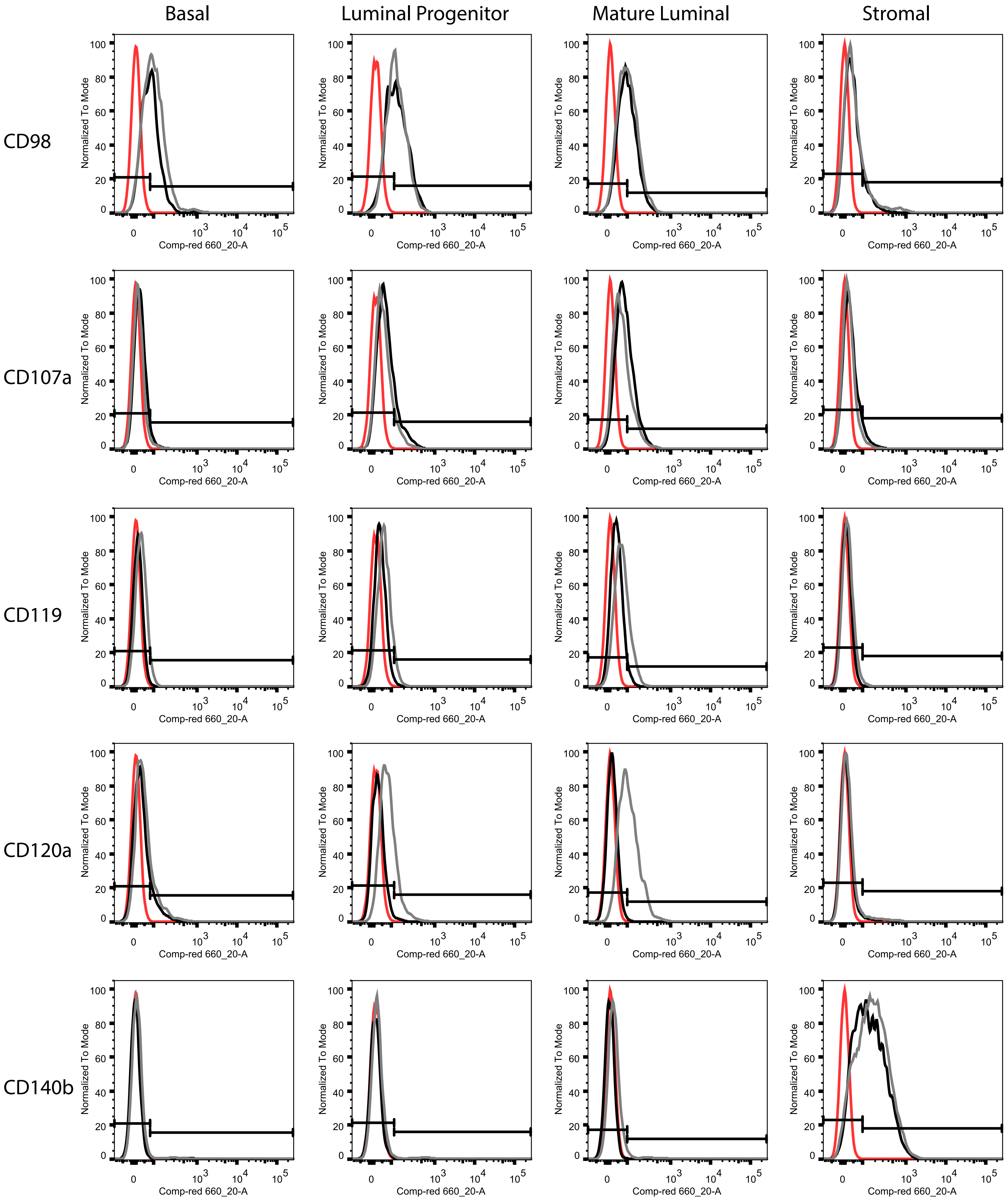

Basal
Luminal Progenitor
Mature Luminal
Stromal
CD98
CD107a
CD119
CD120a
CD140b
Normalized To Mode
Comp-red 660_20-A

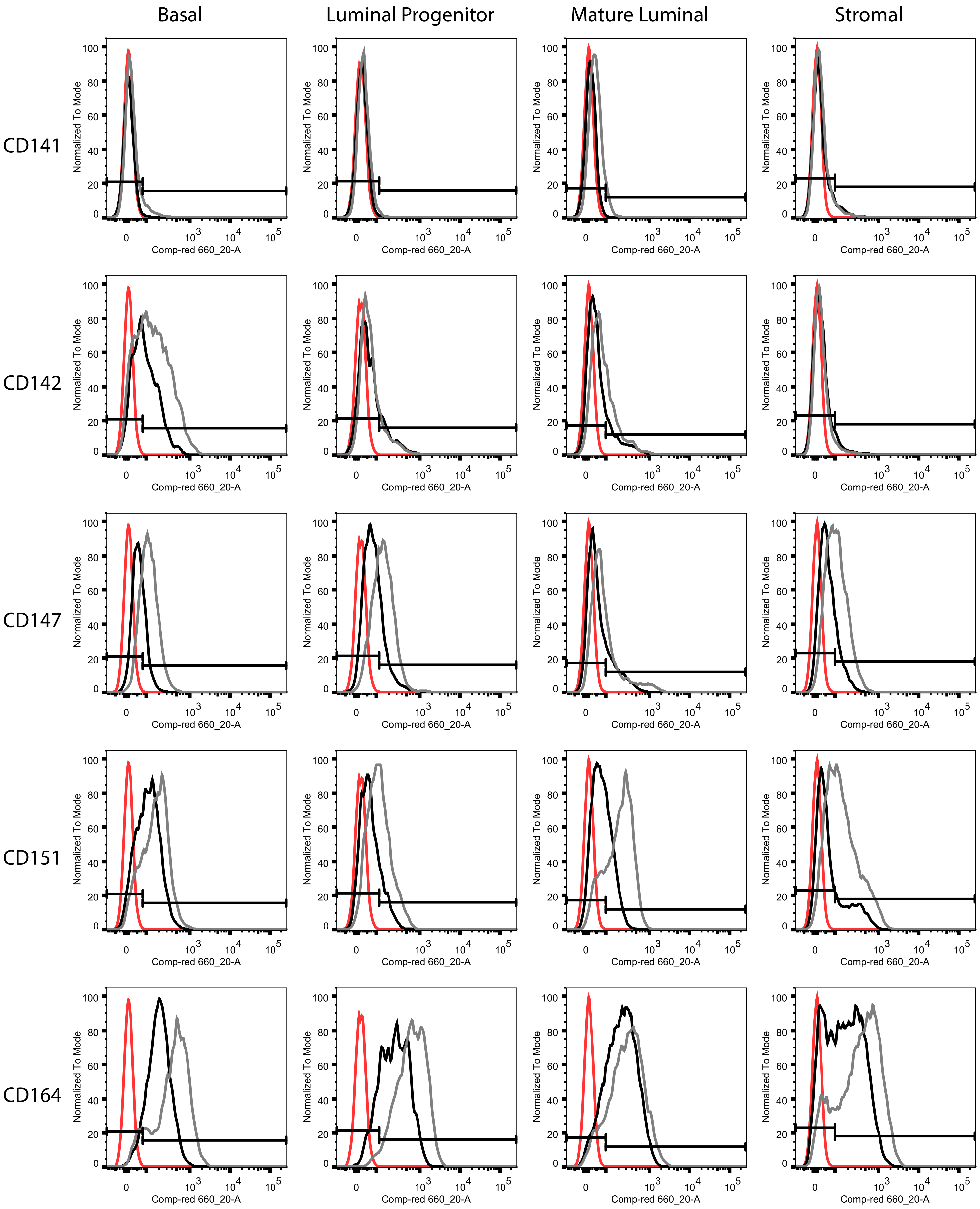

Basal
Luminal Progenitor
Mature Luminal
Stromal
CD141
CD142
CD147
CD151
CD164
Normalized To Mode
Comp-red 660_20-A

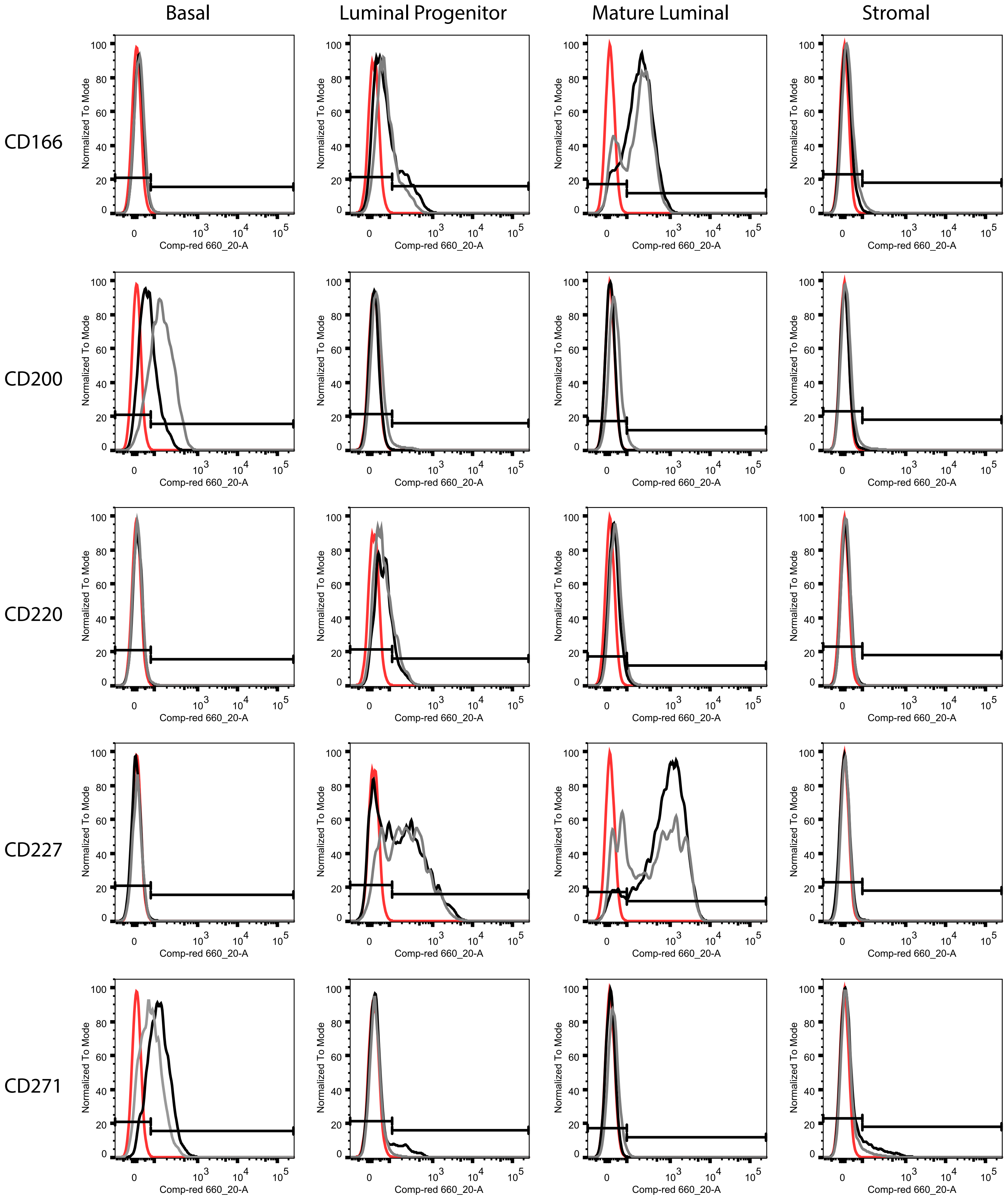
Basal
Luminal Progenitor
Mature Luminal
Stromal
CD166
CD200
CD220
CD227
CD271
Normalized To Mode
Comp-red 660_20-A
0
10^3
10^4
10^5
0
20
40
60
80
100

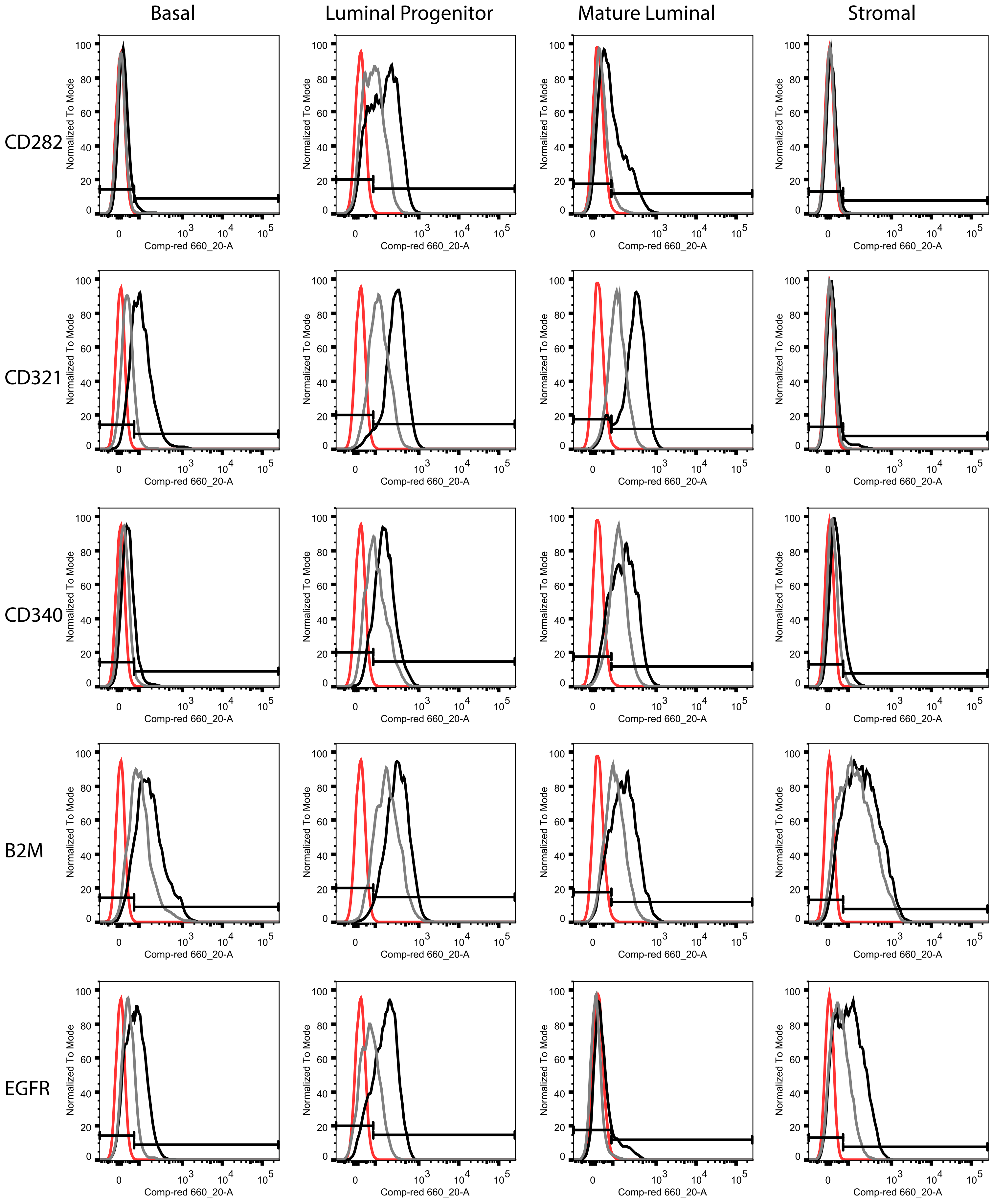

Basal
Luminal Progenitor
Mature Luminal
Stromal
CD282
CD321
CD340
B2M
EGFR
Normalized To Mode
0
20
40
60
80
100
Comp-red 660_20-A
10^3
10^4
10^5

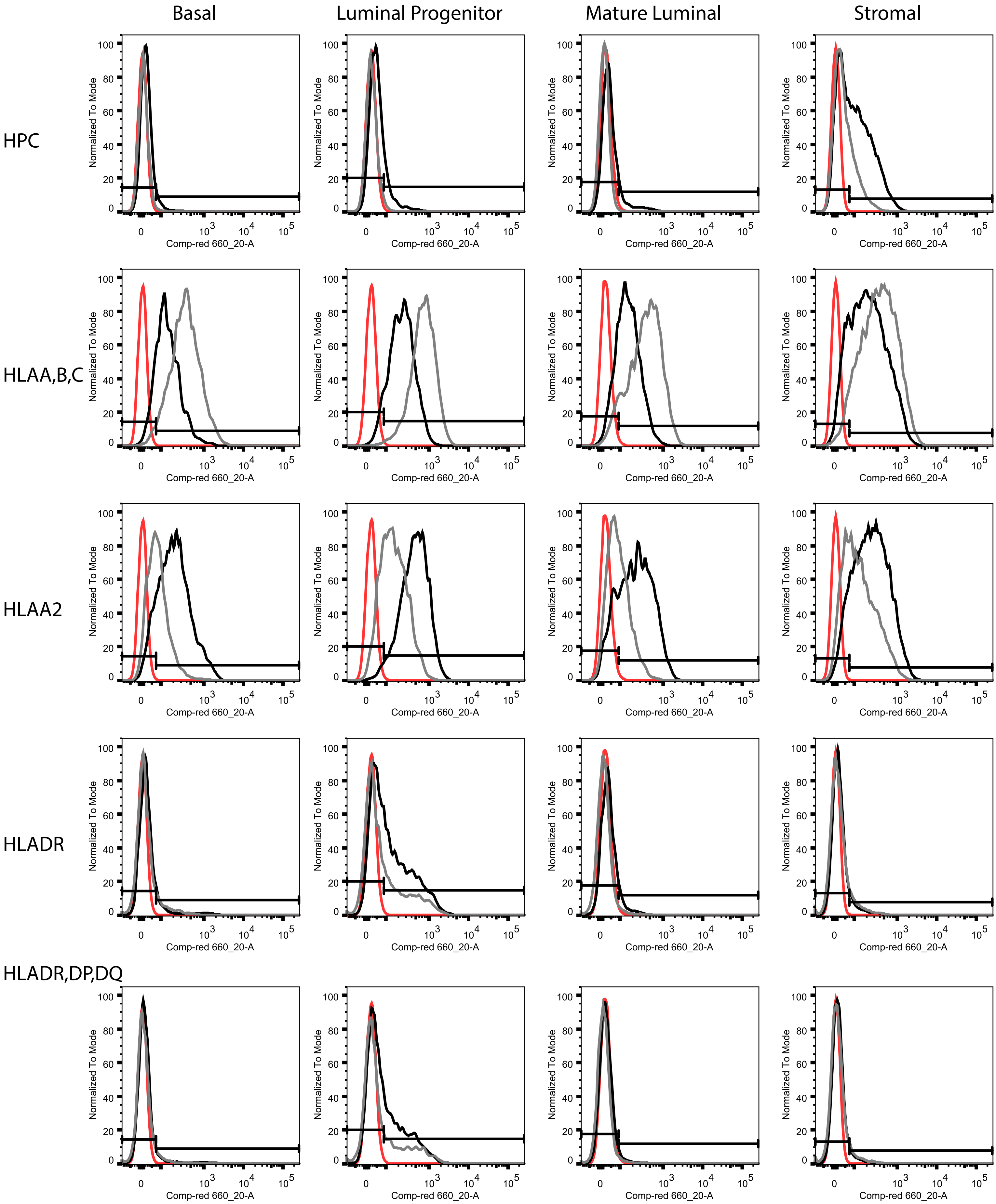
Basal
Luminal Progenitor
Mature Luminal
Stromal
HPC
HLAA,B,C
HLAA2
HLADR
HLADR,DP,DQ
Normalized To Mode
Comp-red 660_20-A

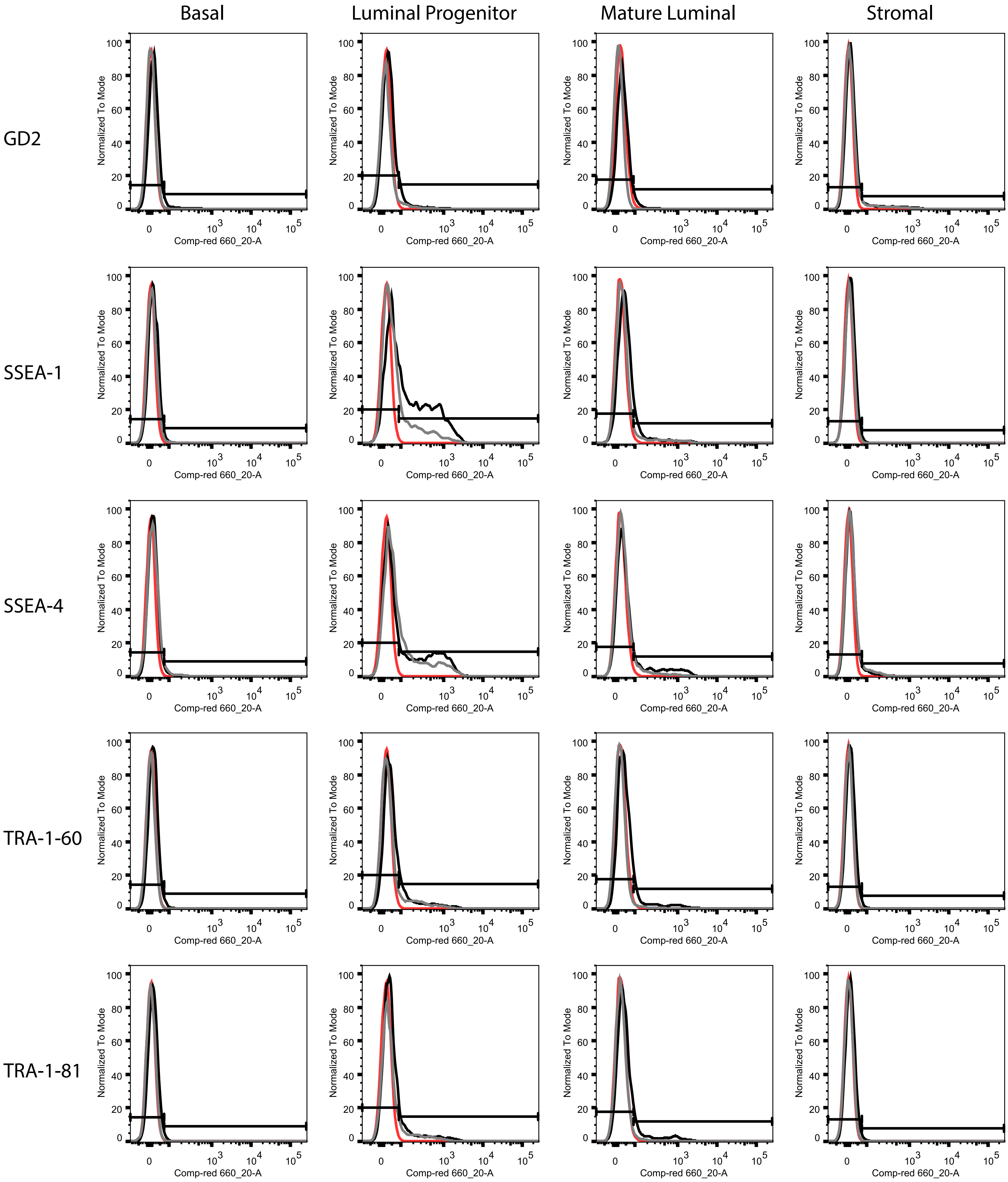
Basal
Luminal Progenitor
Mature Luminal
Stromal
GD2
SSEA-1
SSEA-4
TRA-1-60
TRA-1-81
Normalized To Mode
Comp-red 660_20-A
0
20
40
60
80
100
10^3
10^4
10^5

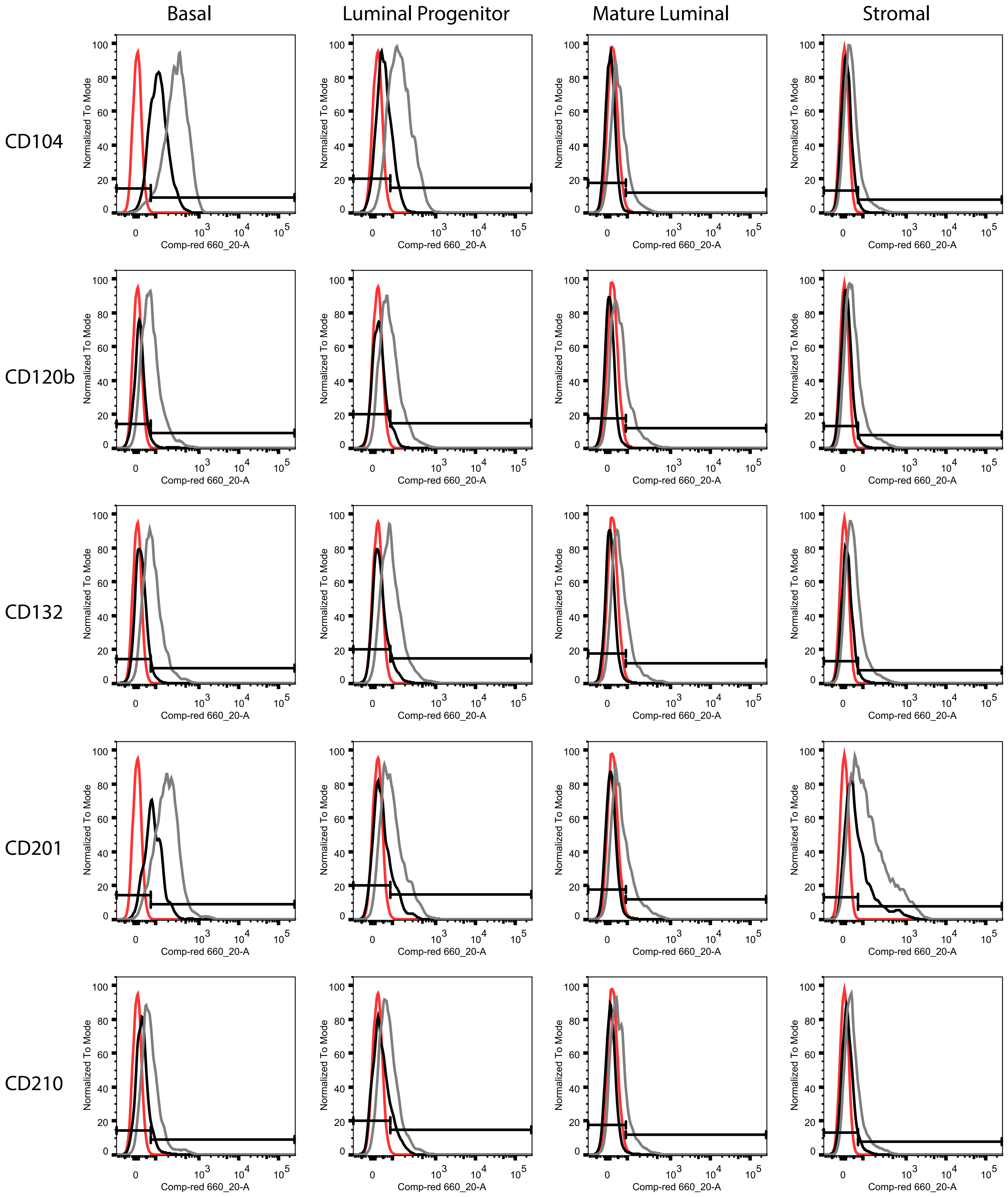
Basal
Luminal Progenitor
Mature Luminal
Stromal
CD104
CD120b
CD132
CD201
CD210
Normalized To Mode
Comp-red 660_20-A
0
20
40
60
80
100
10^3
10^4
10^5

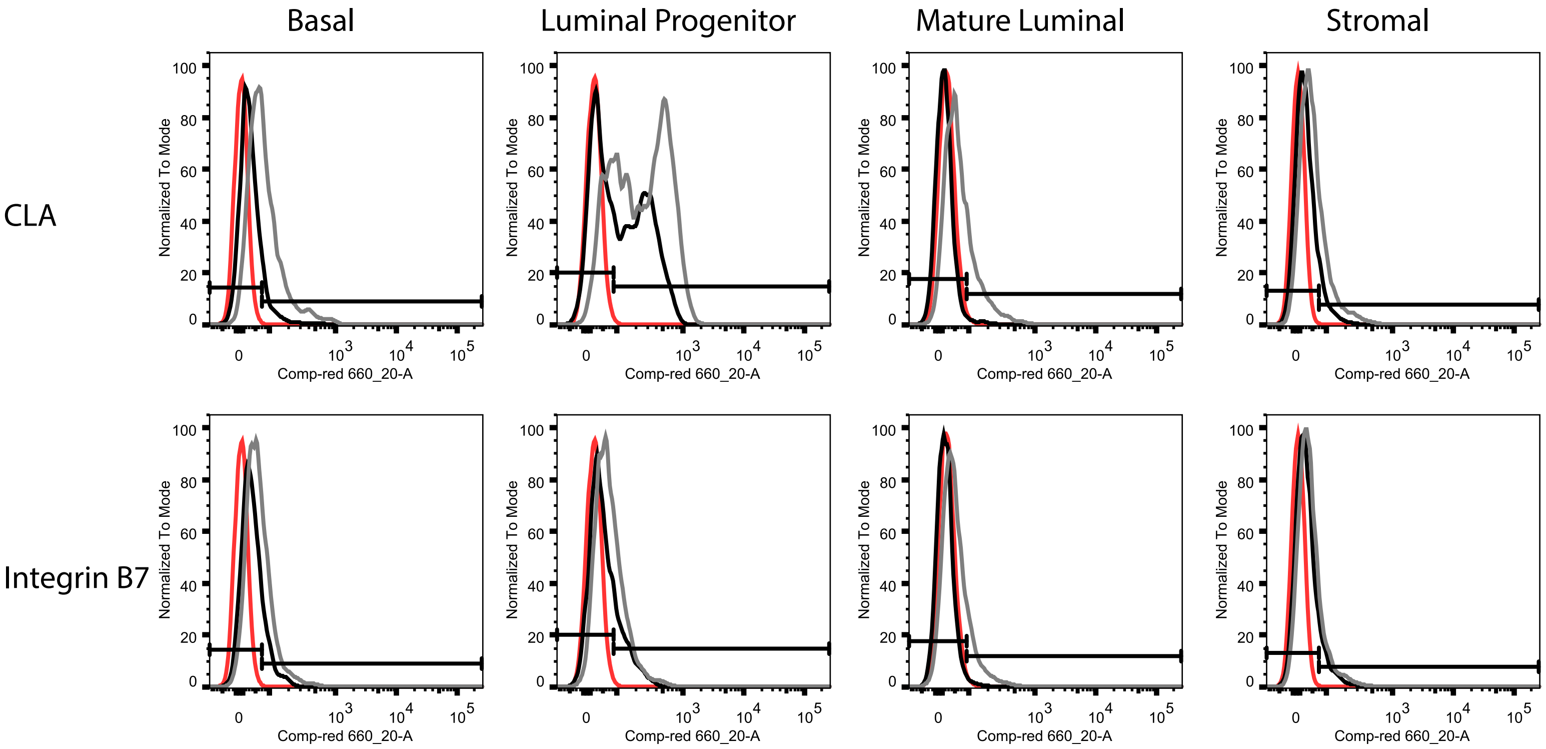
Basal
Luminal Progenitor
Mature Luminal
Stromal
CLA
Integrin B7
Normalized To Mode
Comp-red 660_20-A
